# Author Correction: Reliability of vegetation resilience estimates depends on biomass density

**DOI:** 10.1038/s41559-024-02410-y

**Published:** 2024-04-10

**Authors:** Taylor Smith, Niklas Boers

**Affiliations:** 1 Institute of Geosciences, Universität Potsdam, Potsdam, Germany; 2 Earth System Modelling, School of Engineering and Design, Technical University of Munich, Munich, Germany; 3 Potsdam Institute for Climate Impact Research, Potsdam, Germany; 4 Department of Mathematics and Global Systems Institute, University of Exeter, Exeter, UK

**Keywords:** Ecology, Ecosystem ecology, Climate-change ecology

Correction to: *Nature Ecology & Evolution* 10.1038/s41559-023-02194-7, published online 14 September 2023.

It was recently brought to the authors’ attention that there was an error in the code to produce the kernel normalized difference vegetation index (kNDVI) hosted on Zenodo (https://zenodo.org/records/7550255)—a simple instead of hyperbolic tangent was used to create the kNDVI data from MODIS NDVI data. In order to correct this error, we have rerun all analyses that involved kNDVI using the corrected formula; the formula has also been corrected on the Zenodo repository.

While there are certainly differences between the original data set used in our analysis and the corrected kNDVI data, they do not impact the overall conclusions of our research. None of the statements we make about the kNDVI specifically or MODIS vegetation indices generally are changed by our updated analysis of the data. For some land covers, the correlation between variance and autocorrelation improves, and for others it becomes worse; there are no substantial changes in the global spatial pattern of kNDVI restoring rates (λ) or its trends. The percentages of pixels showing a resilience gain/loss for kNDVI change only slightly (Kendall-Tau: resilience gain changes from 21% to 19.2%, no change in resilience loss; linear trend: resilience gain changes from 21.2% to 19.4%, resilience loss change from 24.3% to 24.5%).

In order to issue a thorough correction, we provide updated versions of each figure in which kNDVI data was displayed. The figures (or panels) that are replaced are noted in the captions of each figure in the Supplementary Information accompanying this article.

### Supplementary information

Original and corrected figures.

## Data Availability

Python scripts to deseason/detrend and export MODIS vegetation data, as well as code to reproduce the synthetic data used in this study, can be found on Zenodo^1^. The code has been updated to use the correct kNDVI formula.
