# Supplementary material for: Reliability of vegetation resilience estimates depends on biomass density
Source: Nat Ecol Evol. 2023 Sep 14;7(11):1799–808. doi: 10.1038/s41559-023-02194-7 (PMC10627832; doi:10.1038/s41559-023-02194-7)
Supplement: Supplementary file 1 — Supplementary Figs. 1–13. [file 41559_2023_2194_MOESM1_ESM.pdf]

nature ecology & evolution

Article https://doi.org/10.1038/s41559-023-02194-7

Reliability of vegetation resilience estimates depends on biomass density

In the format provided by the authors and unedited

Contents

Comparing Data Processing Schemes **2**

The Reliability of Resilience Estimates **6**

Resilience Trends **9**

Methods and Data **11**

Comparing Data Processing Schemes

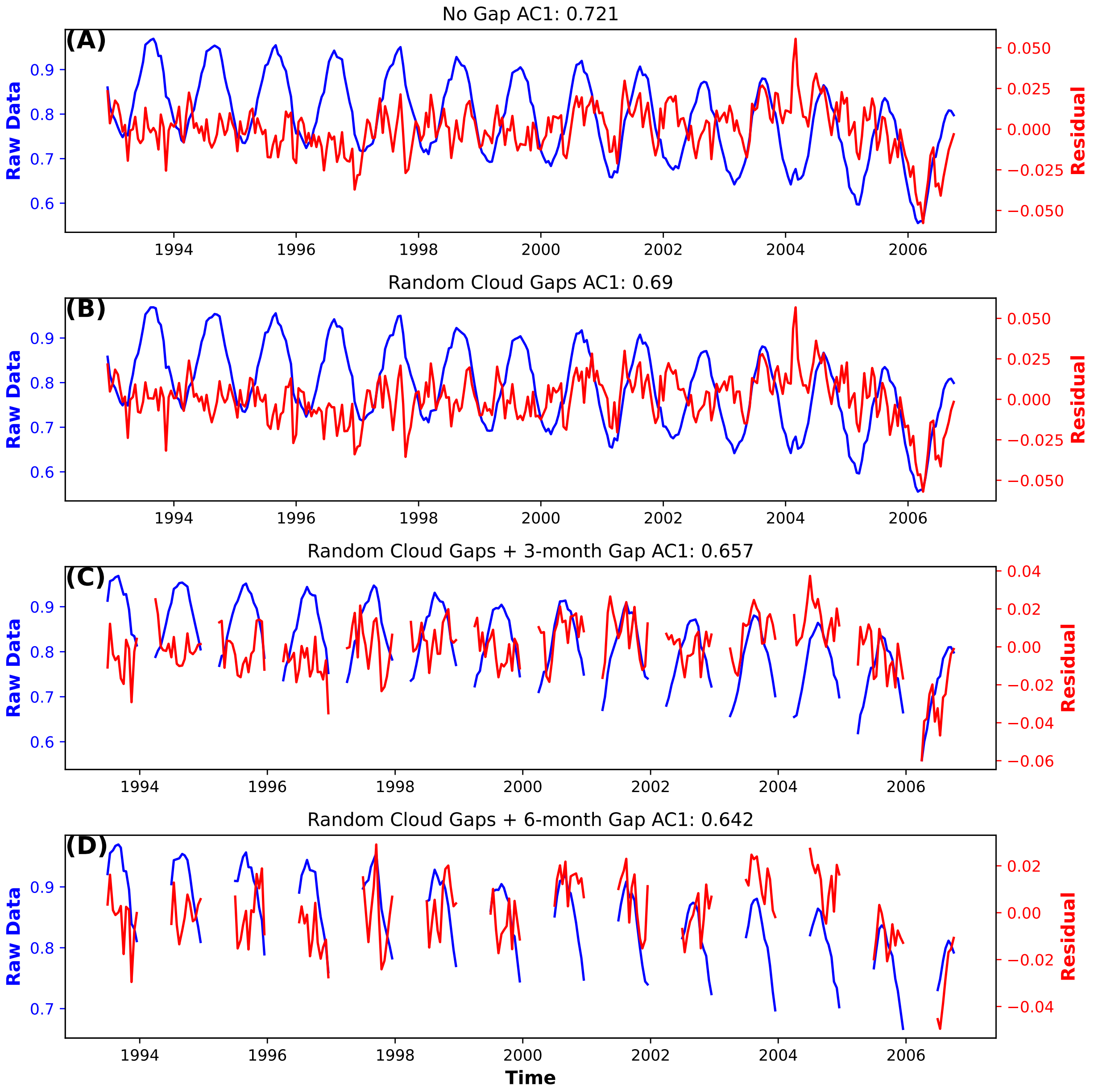

Supplementary Figure S1: Example time series (blue lines) and residual (red lines) created by harmonic deseasoning and rolling-window detrending (Methods). Larger gaps are added from top to bottom, as listed on plot titles. Lag-1 autocorrelation (AC1) for each residual (red) series also listed on chart titles.

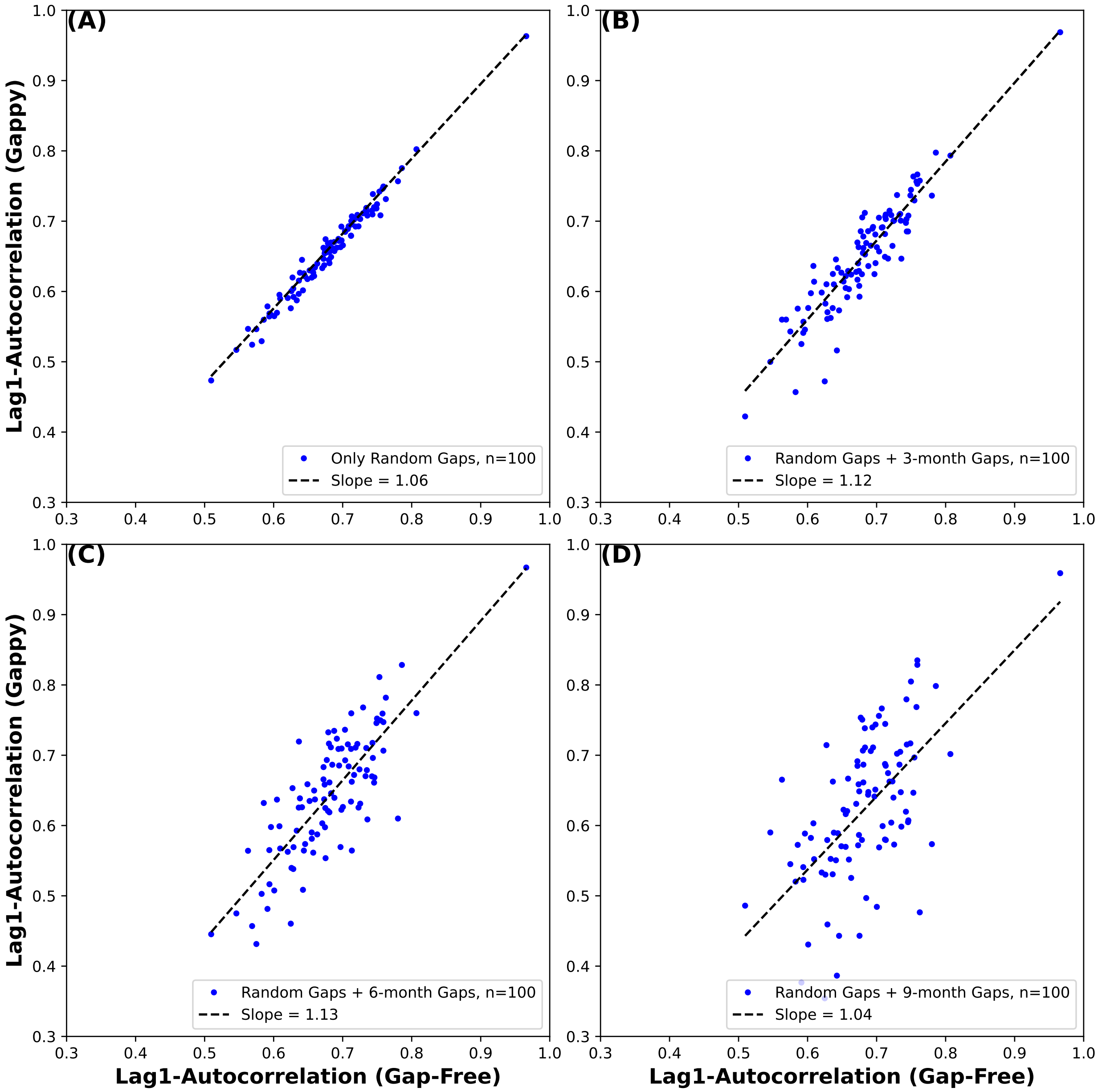

Supplementary Figure S2: Impacts of data gaps on long-term lag-one autocorrelation estimates over 100 simulations. All x-axes are gap-free data, with different sizes of gaps shown in each panel as the y-axis. (A) Random gaps (i.e., clouds), random gaps and additional (B) 3-month-long gaps each year, (C) 6-month-long gaps, and (D) 9-month-long gaps (Methods, cf. Figure S1). Slopes calculated using Sen's method.

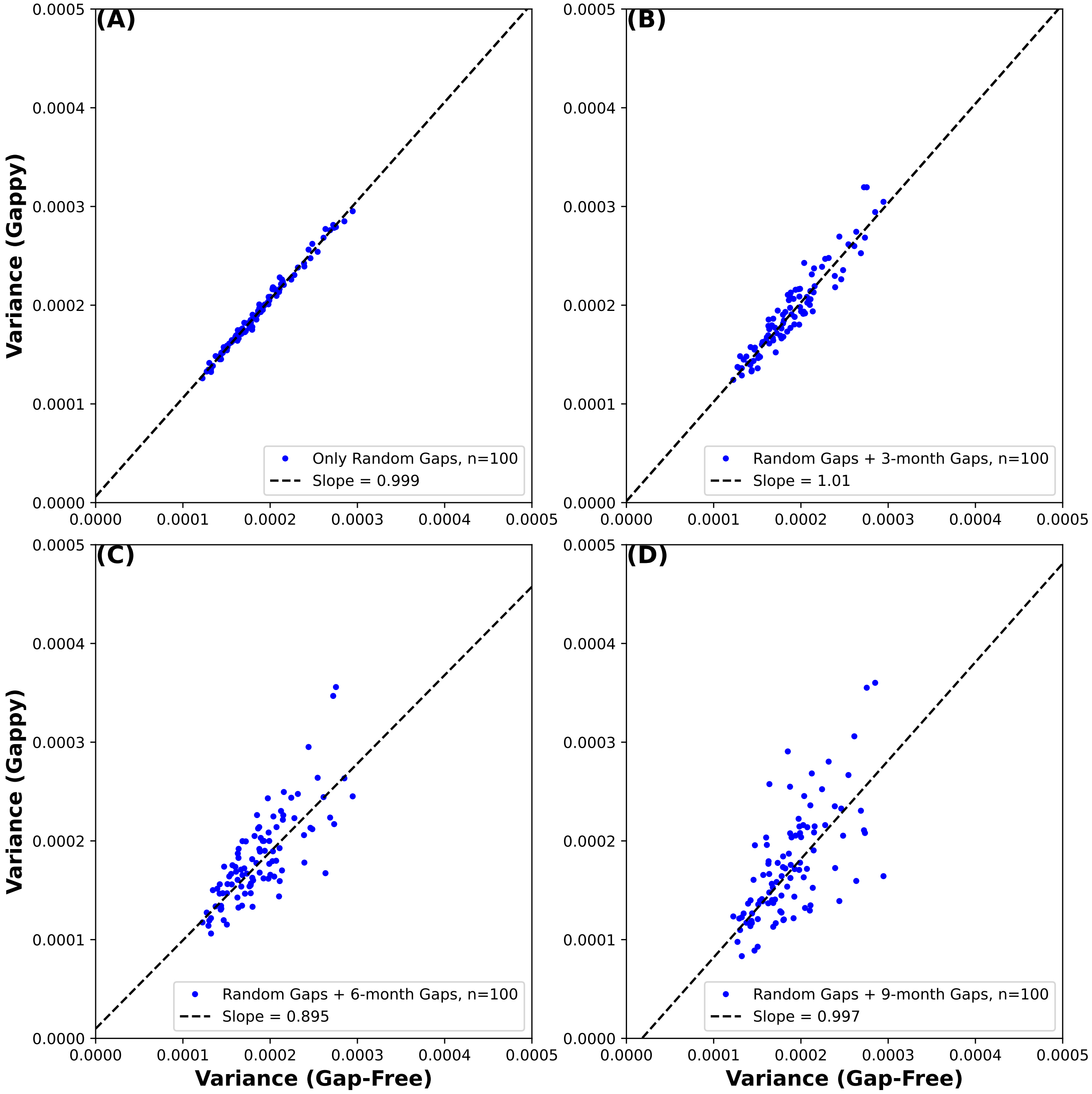

Supplementary Figure S3: Impacts of data gaps on long-term variance estimates over 100 simulations. All x-axes are gap-free data, with different sizes of gaps shown in each panel as the y-axis. (A) Random gaps (i.e., clouds), random gaps and additional (B) 3-month-long gaps each year, (C) 6-month-long gaps, and (D) 9-month-long gaps (Methods, cf. Figure S1). Slopes calculated using Sen's method.

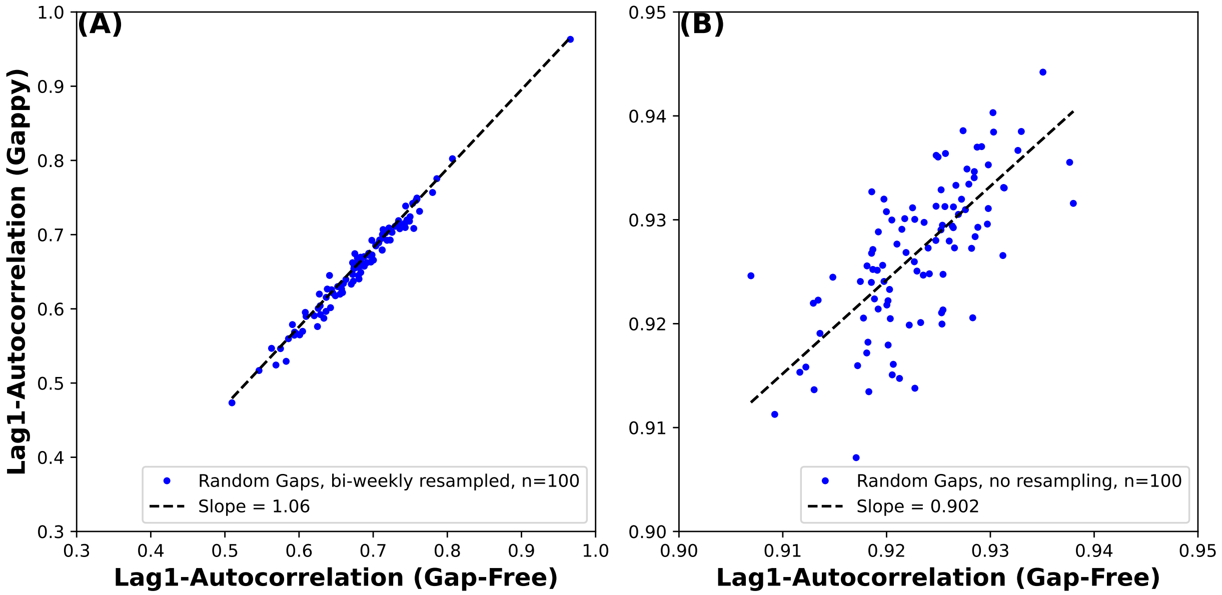

Supplementary Figure S4: Influence of temporal aggregation on AC1 estimates. Both plots have 50% of daily data removed randomly. (A) Data is averaged every two weeks, and (B) no temporal averaging. Both plots show comparison between gappy and gap-free data, indicating that (1) temporal resampling limits the influence of short gaps, and (2) even with randomly distributed gaps, the relationship between gappy and gap-free AC1 remains close to 1:1. Slopes calculated using Sen's method.

2 The Reliability of Resilience Estimates

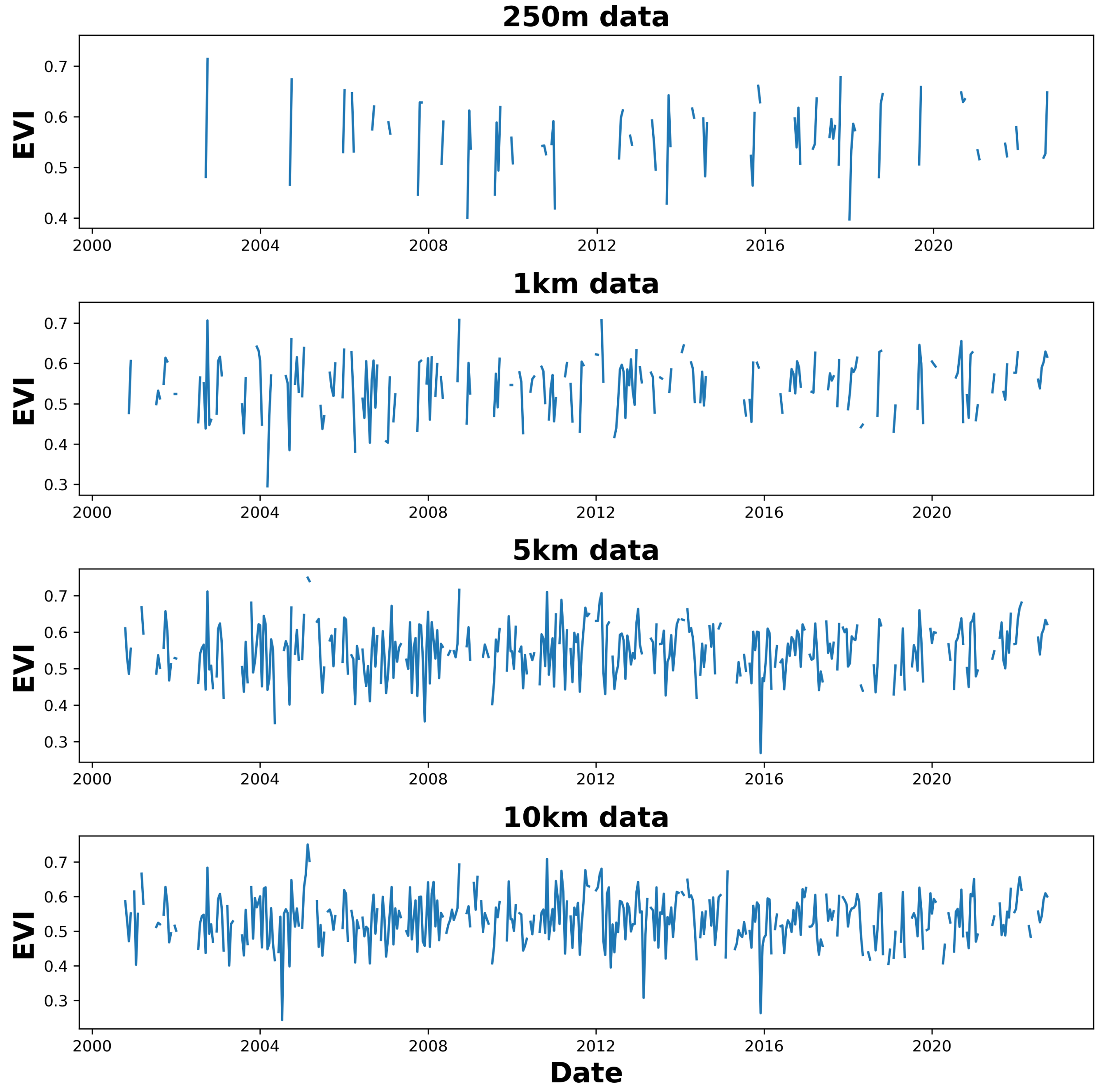

Supplementary Figure S5: Single location EVI data (lon: -70.3245, lat: -1.1450) in dense vegetation showing time series with negative autocorrelation. This location could not be used successfully for resilience estimation, even after spatial aggregation.

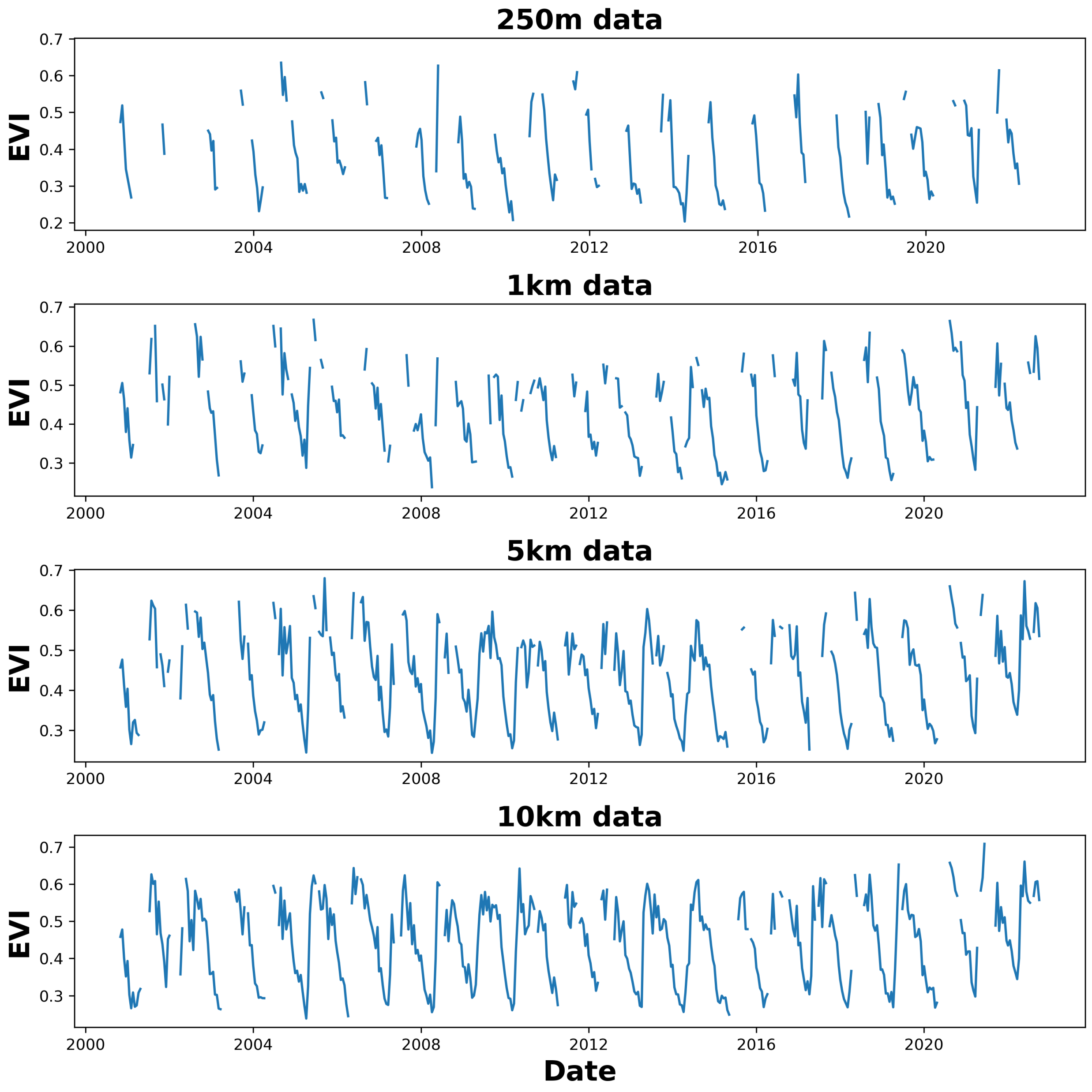

Supplementary Figure S6: Single location EVI data (lon: -68.931, lat: 8.406) in moderate vegetation showing time series with positive autocorrelation. This location could be used successfully for resilience estimation, despite the data gaps. Note the improvements in data density with spatial aggregation.

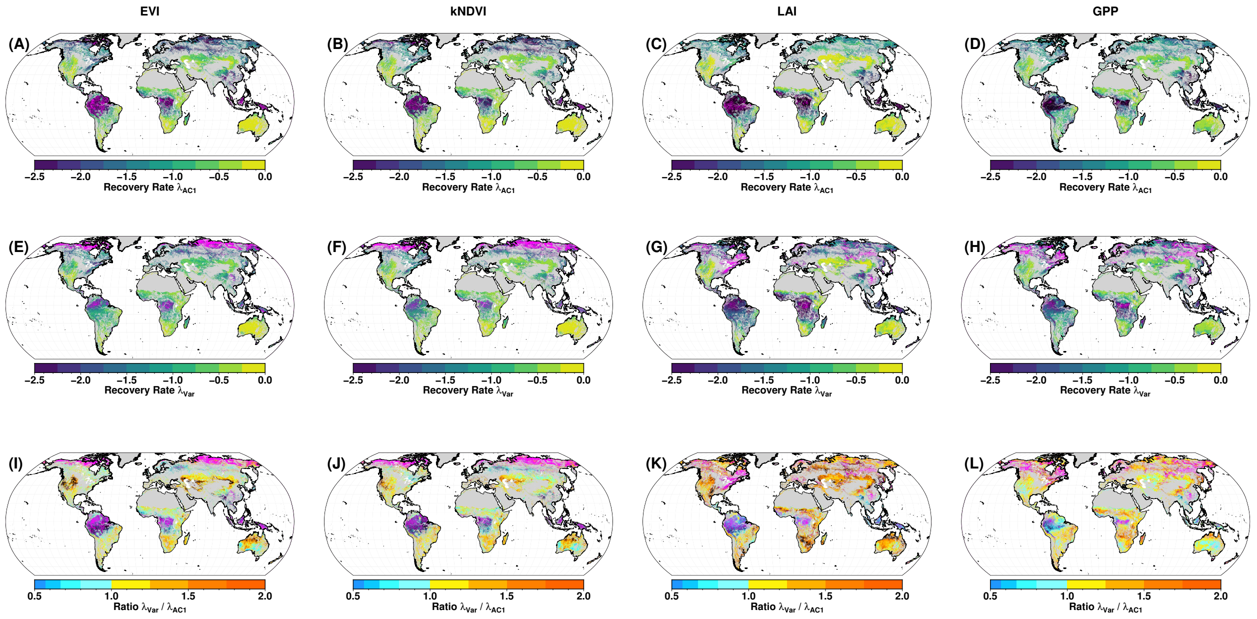

Supplementary Figure S7: Comparison of global λ estimates from AC1/variance, using EVI, kNDVI, LAI, and GPP (see Figure 3). Magenta areas show unconstrained λ estimates (see Methods), black areas have a too-large $\lambda_{Var}/\lambda_{AC1}$ ratio (less than half or above a factor of two).

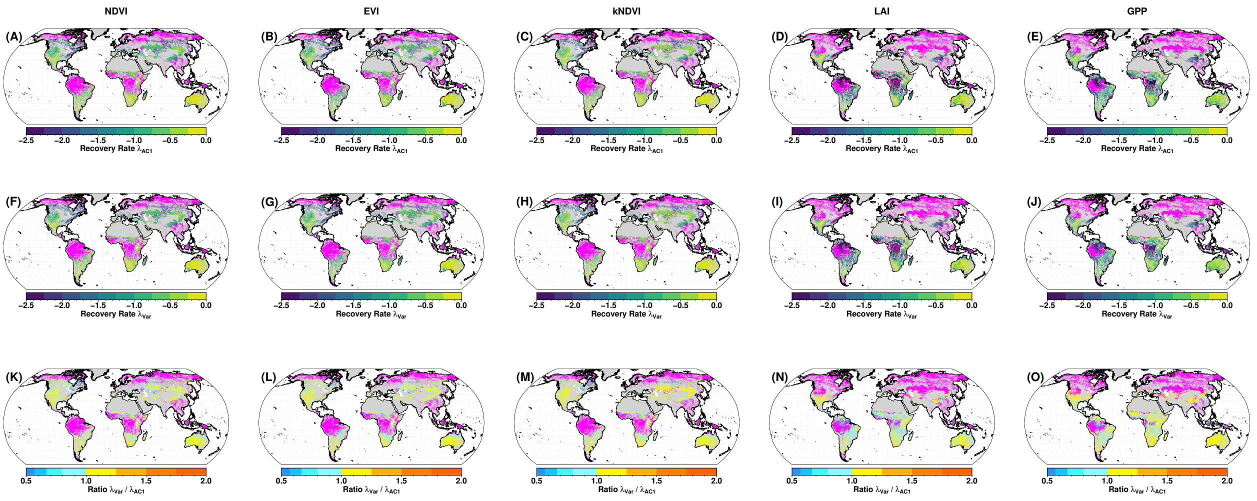

Supplementary Figure S8: Comparison of global λ estimates from AC1/variance, using NDVI, EVI, kNDVI, LAI, and GPP (see Figure 3); data preprocessed using STL. Magenta areas show unconstrained λ estimates (see Methods), black areas have a too-large $\lambda_{Var}/\lambda_{AC1}$ ratio (less than half or above a factor of two).

Resilience Trends

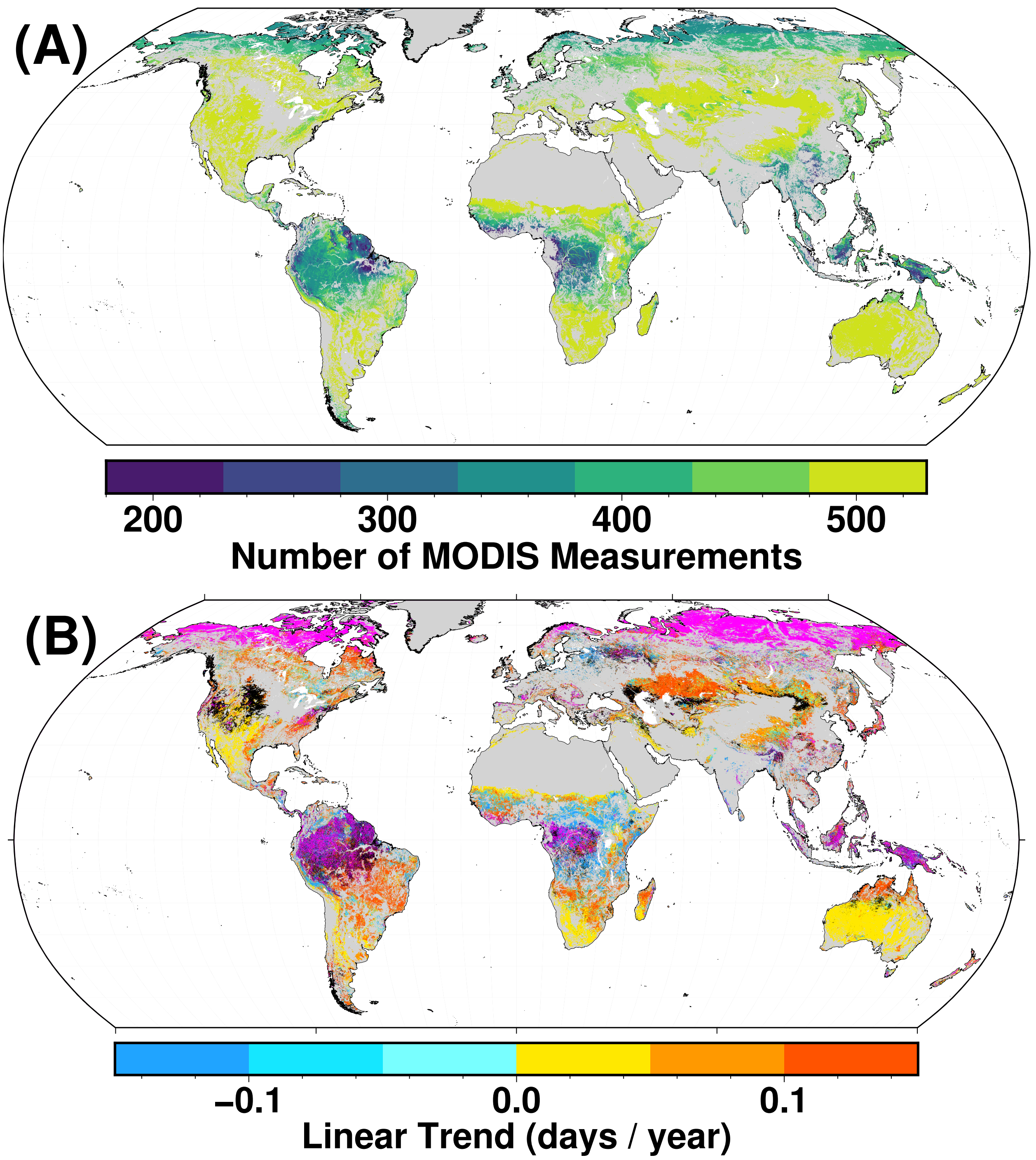

Supplementary Figure S9: (A) Number of NDVI measurements for each 5 km pixel (2000-2022). Grey areas masked for land cover (see Methods). Note the prevalence of gaps in both the tropics and high latitude regions. (B) Trends in data density through time. Only very small changes in data density through time are apparent globally. Black areas masked for high $\lambda_{Var}/\lambda_{AC1}$ ratios (less than half or above a factor of two). Magenta areas indicate undefined λ_{AC1} or λ_{Var} estimates.

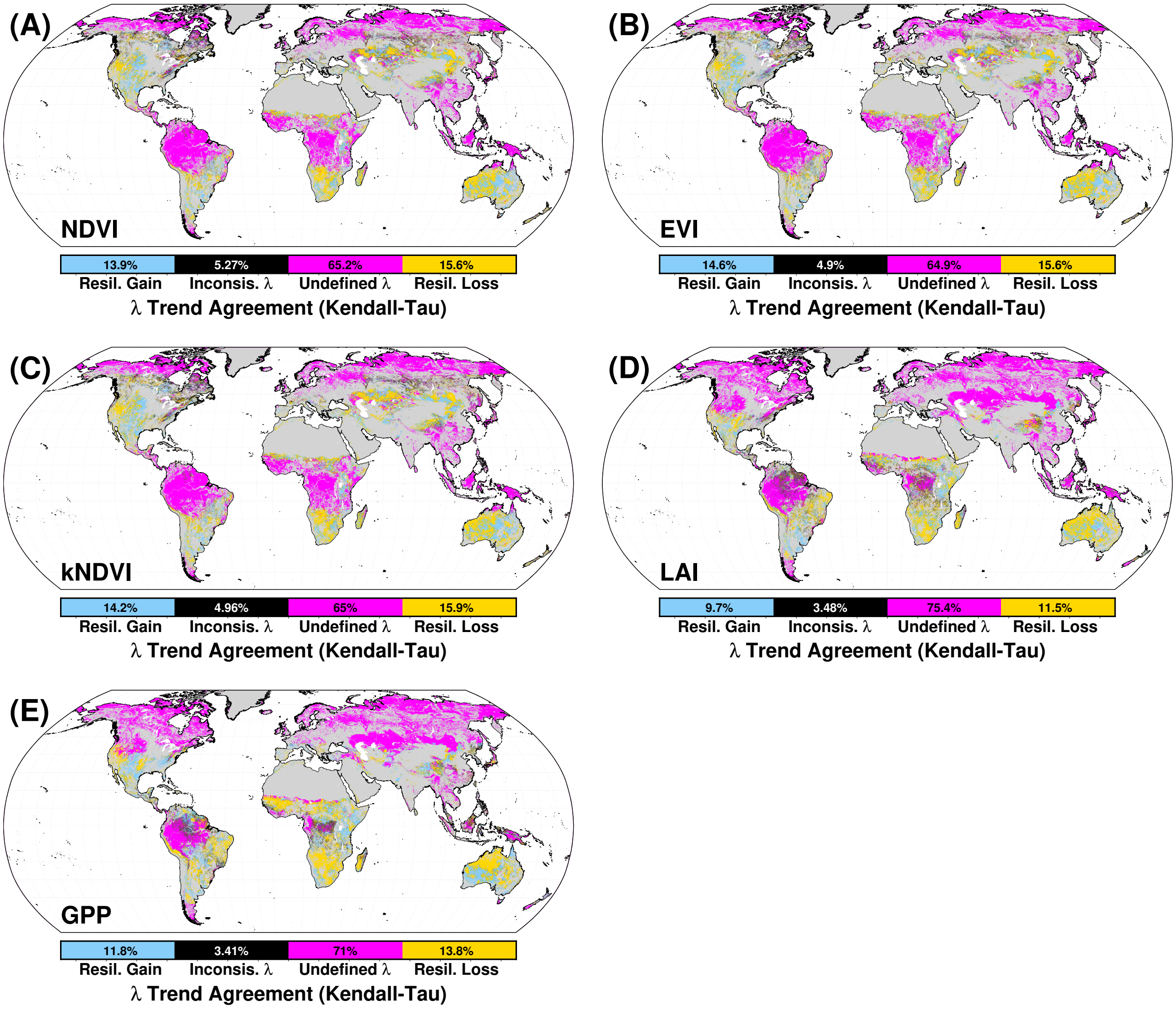

Supplementary Figure S10: Global Kendall-Tau trends in resilience across all vegetation indices at 5 km resolution; data preprocessed using STL. Grey areas masked for land cover (see Methods). Areas of agreement between variance- and AC1-based λ marked as resilience gain or loss, others as inconsistent (high $\lambda_{Var}/\lambda_{AC1}$ ratio, black) or undefined λ (magenta).

4 # Methods and Data

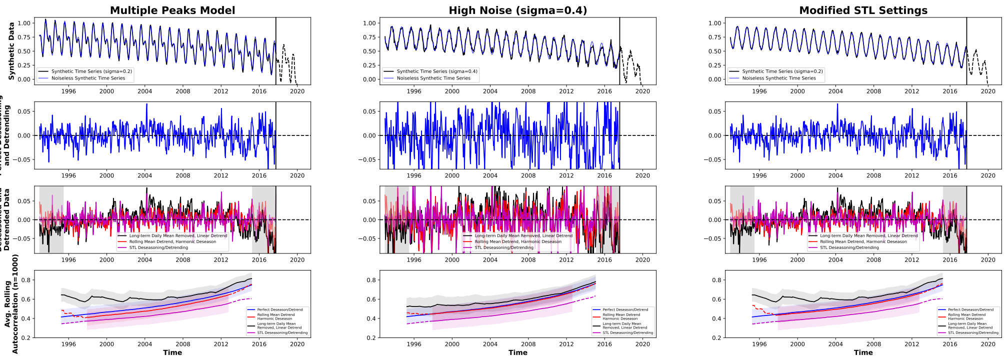

Supplementary Figure S11: Comparison of further synthetic seasonality models (see Figure 1). From the left: multiple seasonal peaks, high noise (sigma=0.4), and modified STL settings (longer seasonal smoother). Python code to reproduce all synthetic data can be found on Zenodo: 10.5281/zenodo.7550255

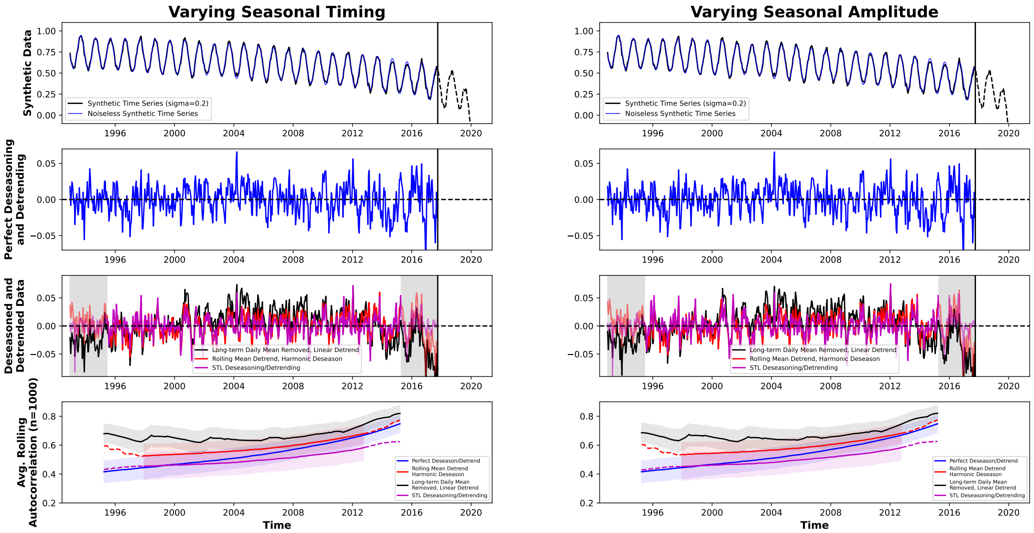

Supplementary Figure S12: Comparison of further synthetic seasonality models (see Figure 1). Left: varying seasonal timing, right: varying seasonal amplitude. Python code to reproduce all synthetic data can be found on Zenodo: 10.5281/zenodo.7550255

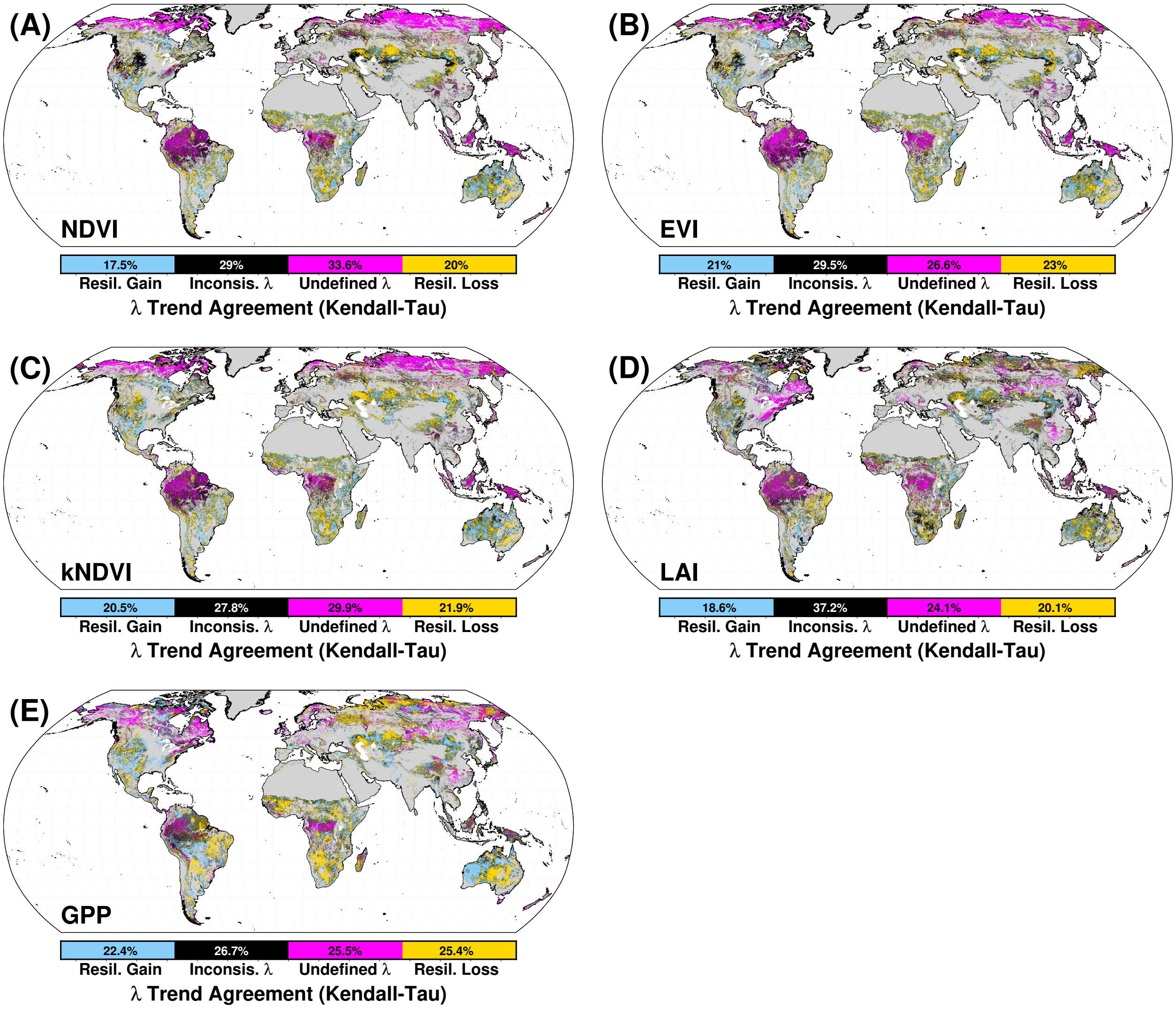

Supplementary Figure S13: Global Kendall-Tau trends in resilience across all vegetation indices at 5 km resolution, over a shorter time period (2004-2017). Grey areas masked for land-cover (see Methods). Areas of agreement between variance- and AC1-based λ marked as resilience gain or loss, others as inconsistent (high $\lambda_{Var}/\lambda_{AC1}$ ratio or trend disagreement, black) or undefined λ (magenta).
