# Supplementary material for: Author Correction: Reliability of vegetation resilience estimates depends on biomass density
Source: Nat Ecol Evol. 2024 Apr 10;8(6):1199. doi: 10.1038/s41559-024-02410-y (PMC11166565; doi:10.1038/s41559-024-02410-y)
Supplement: Supplementary file 1 — Original and corrected figures. [file 41559_2024_2410_MOESM1_ESM.pdf]

Author Correction: Reliability of vegetation resilience estimates depends on biomass density

In the format provided by the authors and unedited

Updated Figures - Main Text

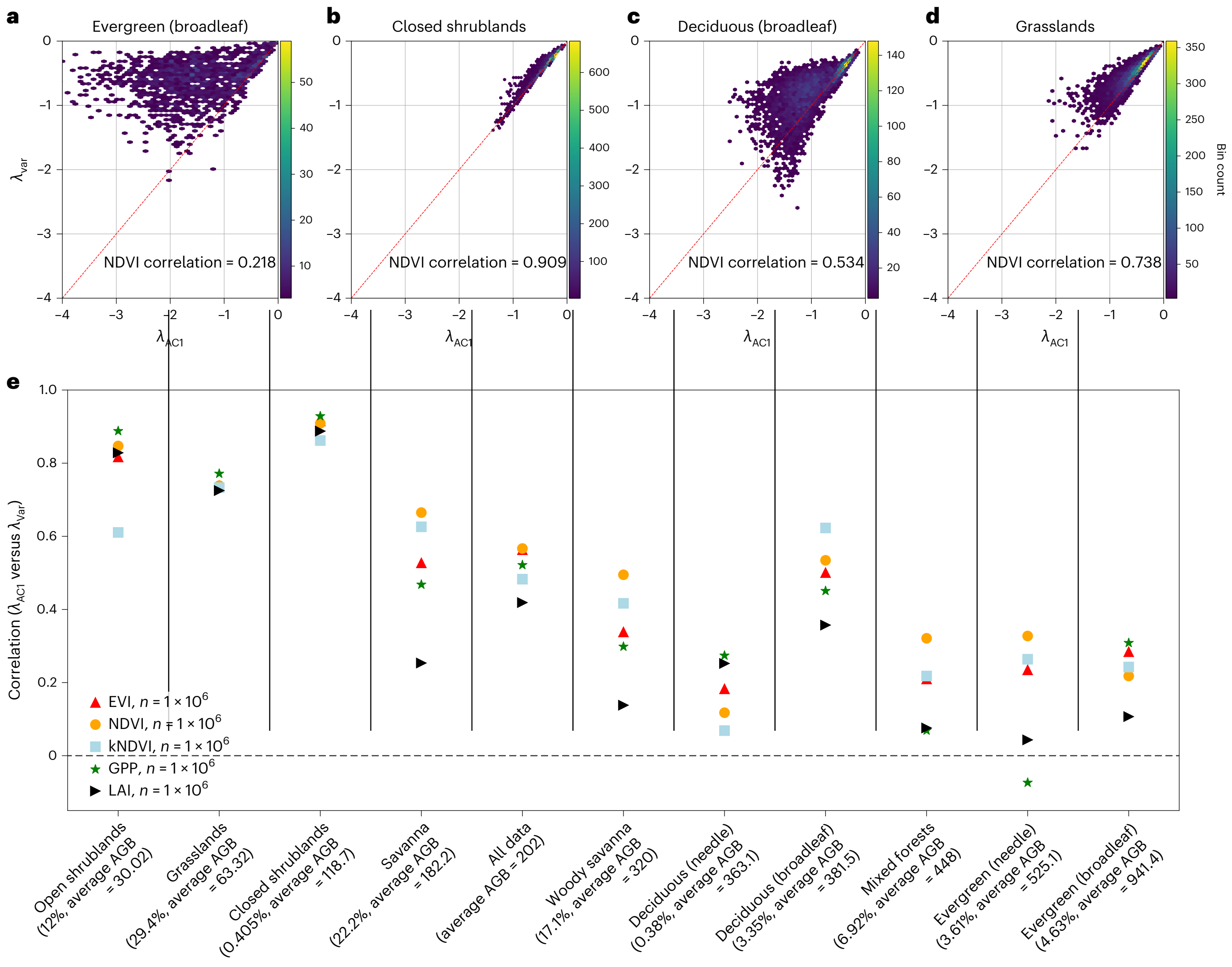

Original Figure 2

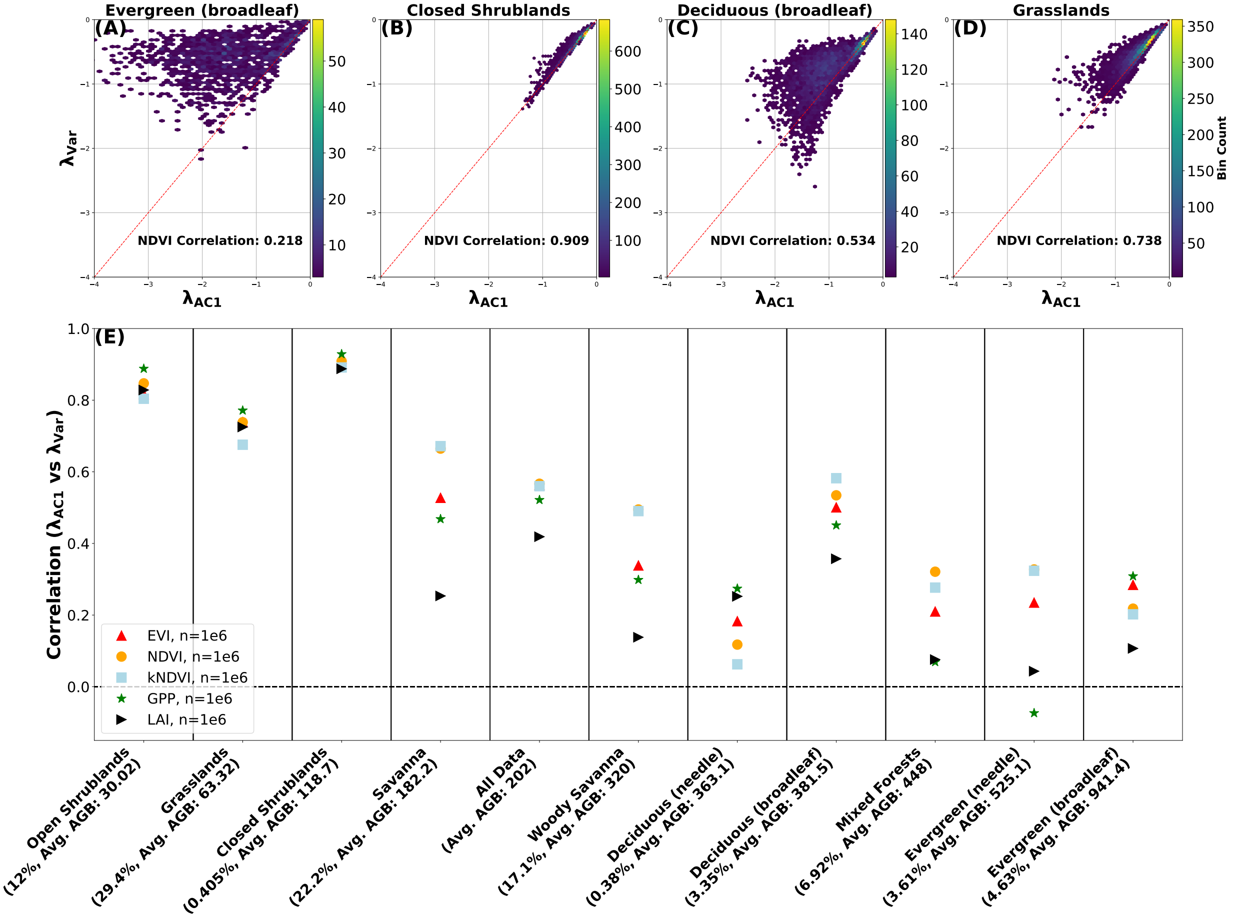

Corrected Figure 2: Relationship between AC1- and variance-based estimates of the recovery rate λ using NDVI data at MODIS-native resolution (250 m). (A) Evergreen Broadleaf (n=10,000), (B) Closed Shrublands (n=10,000), (C) Deciduous Broadleaf (n=10,000), and (D) Grasslands (n=10,000) behave distinctly differently, with Shrublands hewing closest to the expected one-to-one relationship between λ_{AC1} and λ_{Var} (red dashed line). (E) Pearson's correlation for n=100,000 points (n=10,000 for each natural land cover type), compared for all MODIS vegetation indices at native sensor resolution (EVI/NDVI/kNDVI: 250 m, GPP/LAI: 500 m). The kNDVI markers (light blue squares, panel E) have been modified from the original manuscript version (Figure 2).

Updated Figures - Extended Data

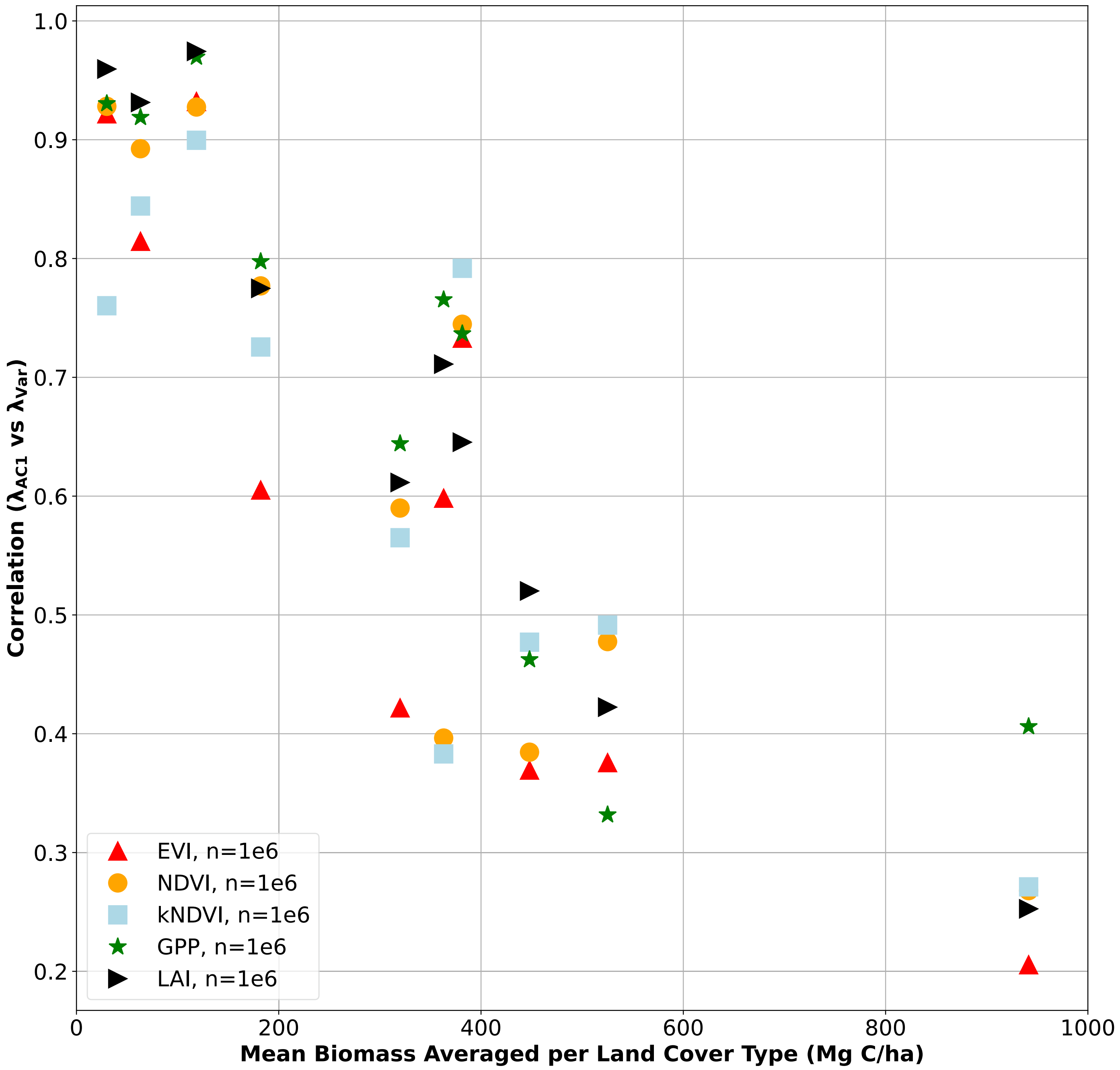

Original Extended Data Figure 1

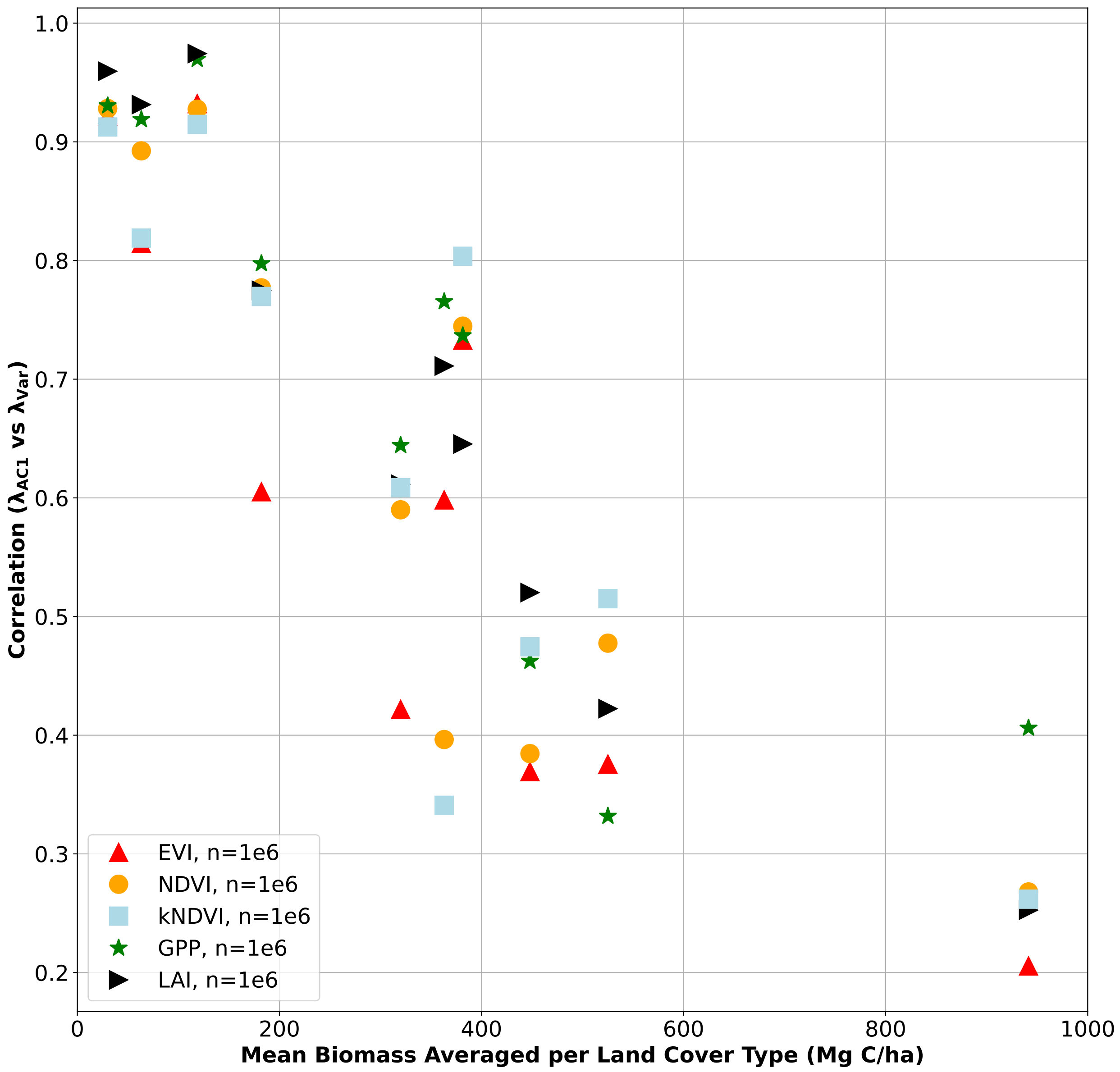

Corrected Figure ED1: Comparison between biomass and λ_{Var}-λ_{AC1} correlation coefficients with a floating x-scale. Shows a strongly linear relationship between correlation and biomass across all indices; n=100,000 for each index. The kNDVI markers (light blue squares) have been modified from the original manuscript version (Extended Data Figure 1).

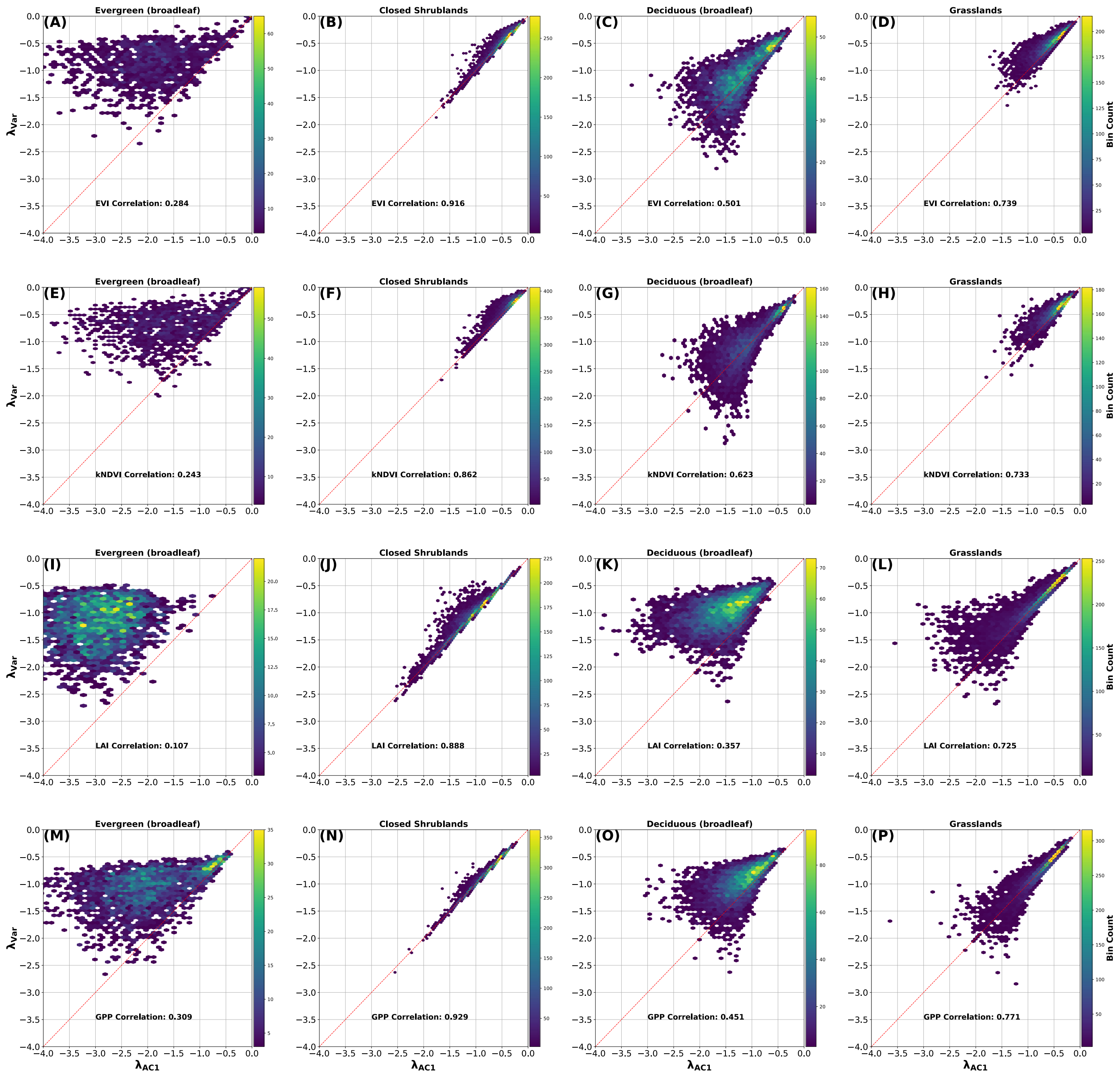

Original Extended Data Figure 2

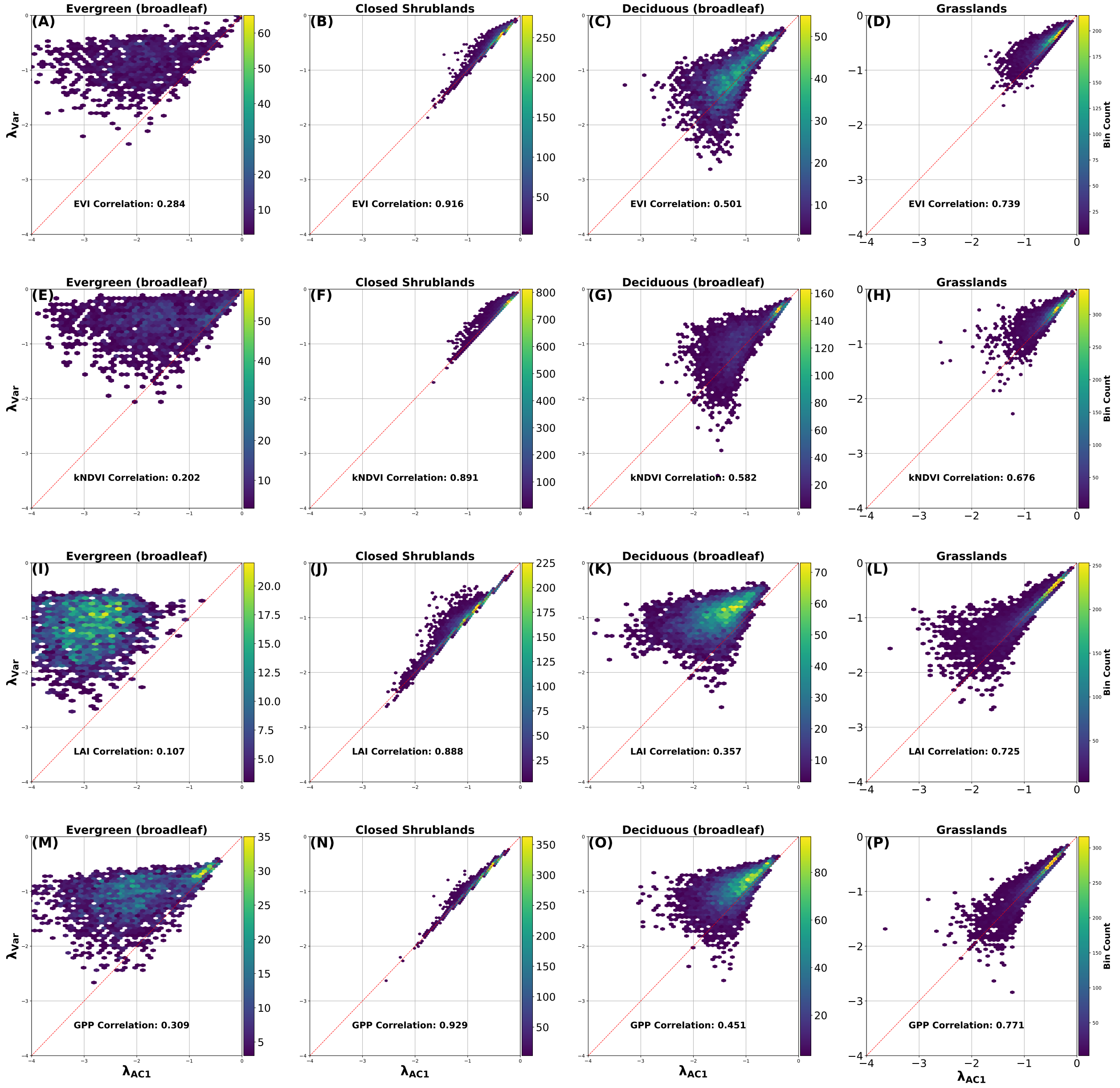

Corrected Figure ED2: Comparison of $\lambda_{Var}/\lambda_{AC1}$ for the same data points (n=10,000 for each individual land cover type). Each row covers one vegetation index, from top to bottom: EVI, kNDVI, LAI, GPP. Correlation coefficients listed on charts, with red 1:1 line shown for reference. Minimum 3 points per bin. Panels E-H have been updated from the original manuscript version (Extended Data Figure 2).

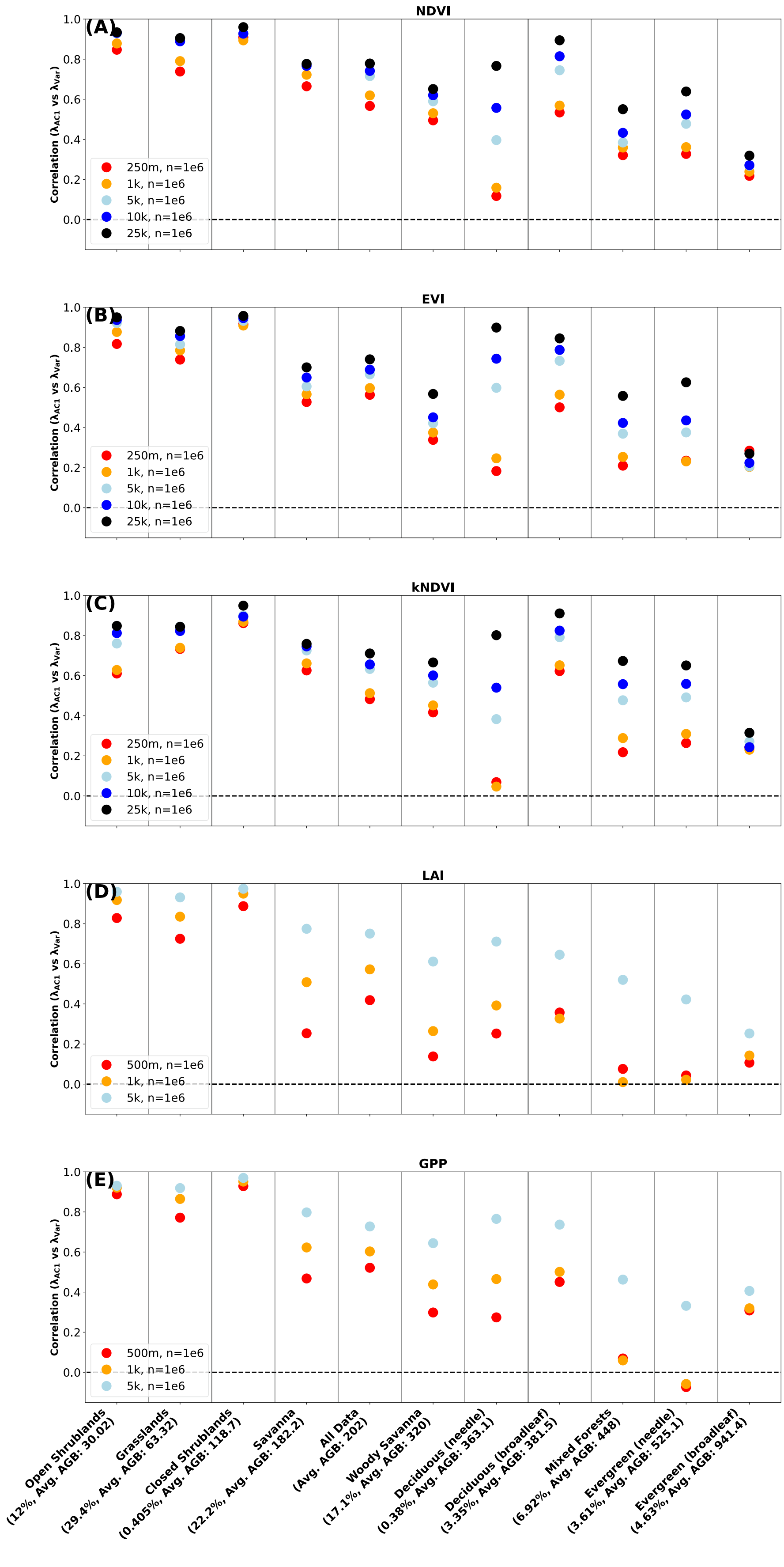

Original Extended Data Figure 3

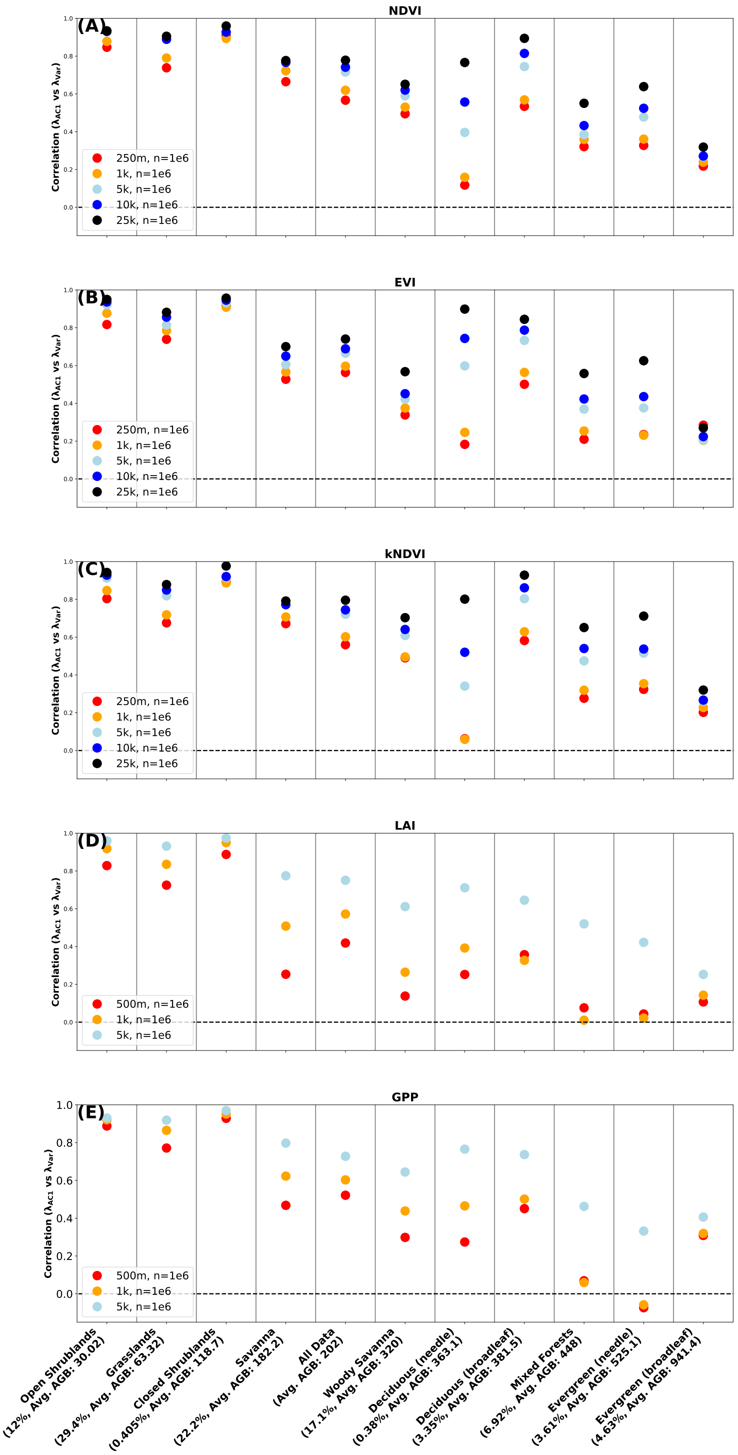

Corrected Figure ED3: Comparison of $\lambda_{AC1}/\lambda_{Var}$ correlation coefficients for the same data points (n=100,000, 10,000 for each land cover type) at different levels of spatial aggregation, across all tested vegetation indices. Land cover types sorted by above-ground biomass (AGB). Each plot covers one vegetation index. 10 km and 25 km data not included for LAI/GPP due to processing constraints. Panel C has been updated from the original manuscript version (Extended Data Figure 3).

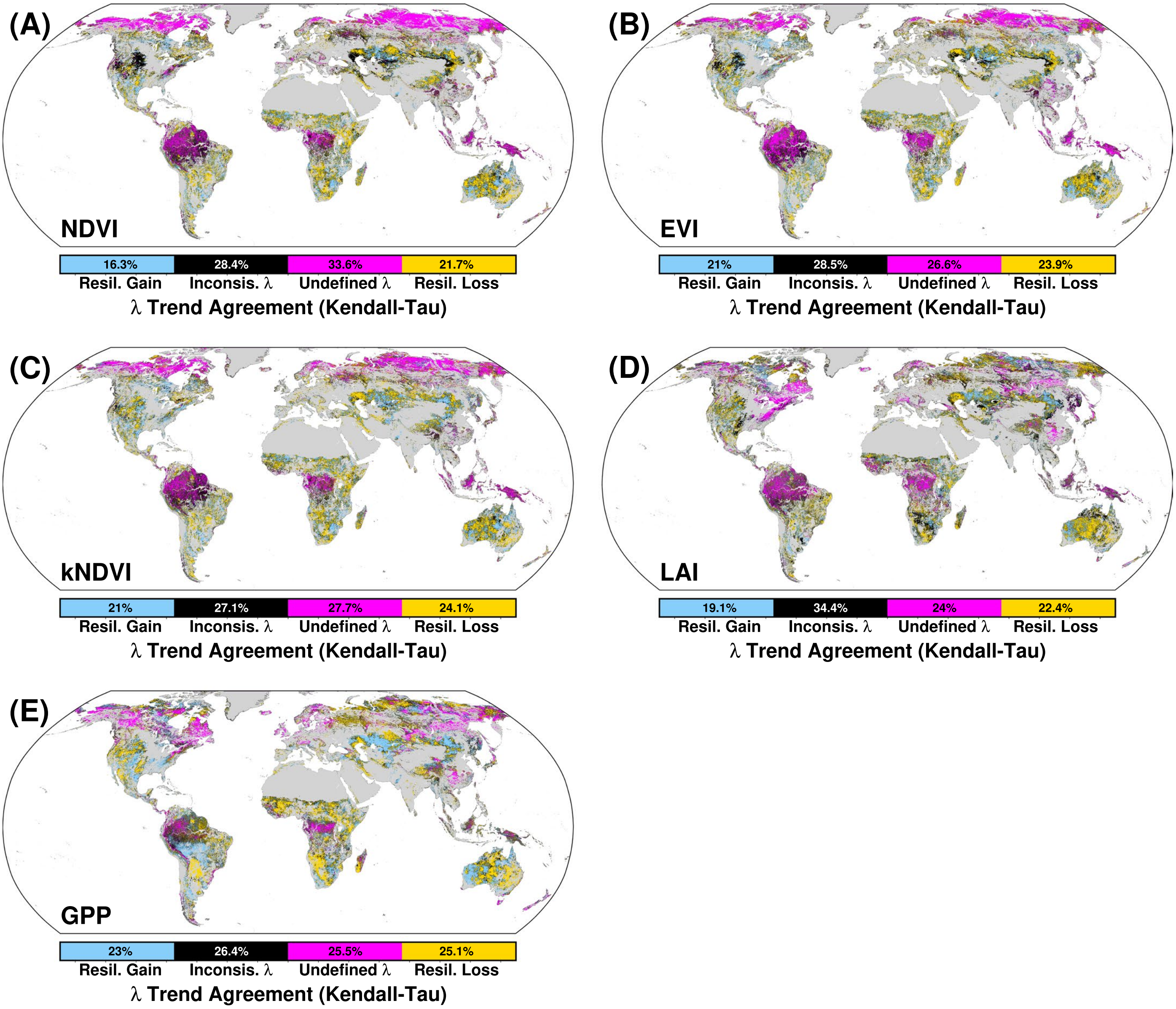

Original Extended Data Figure 4

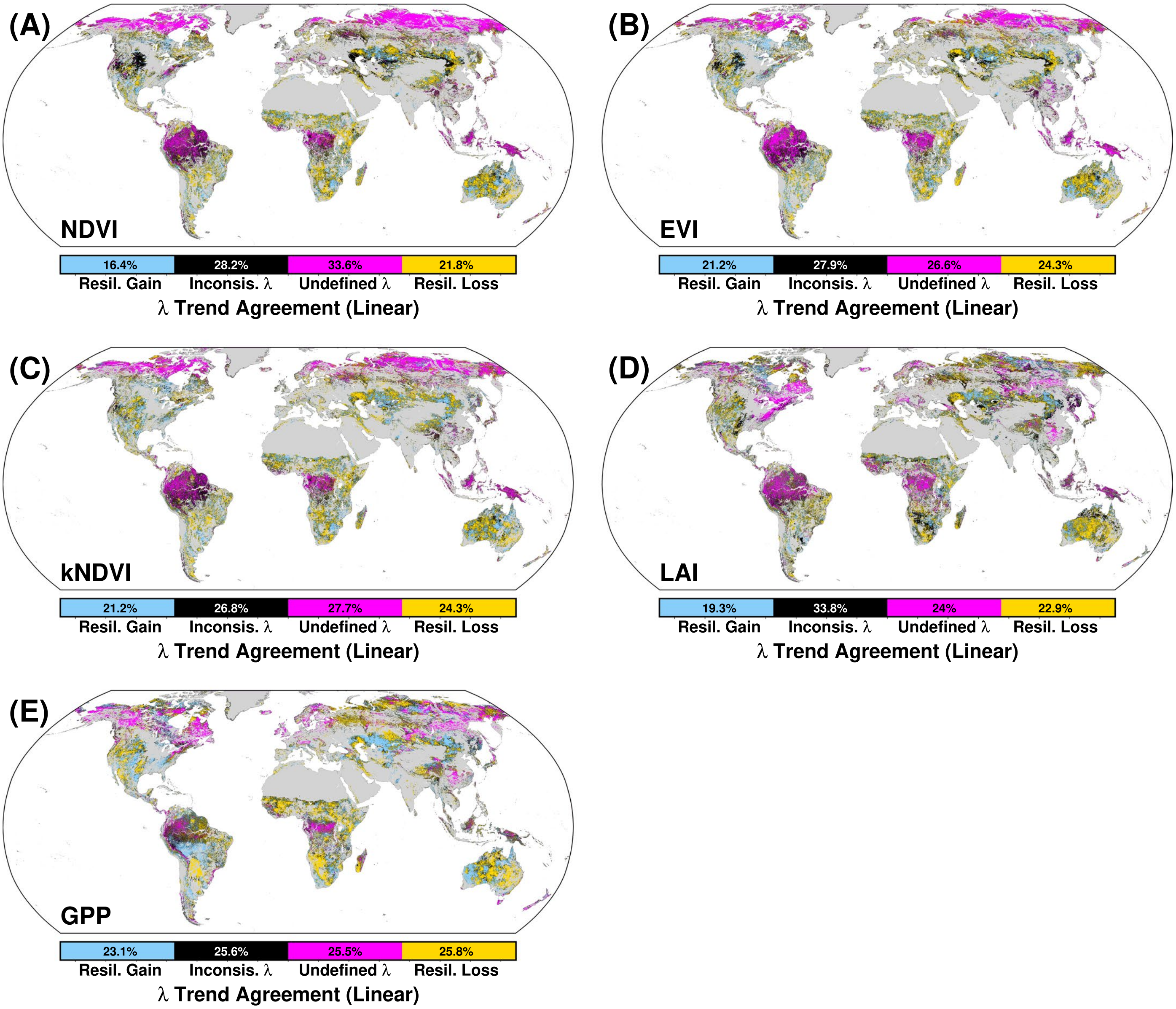

Original Extended Data Figure 5

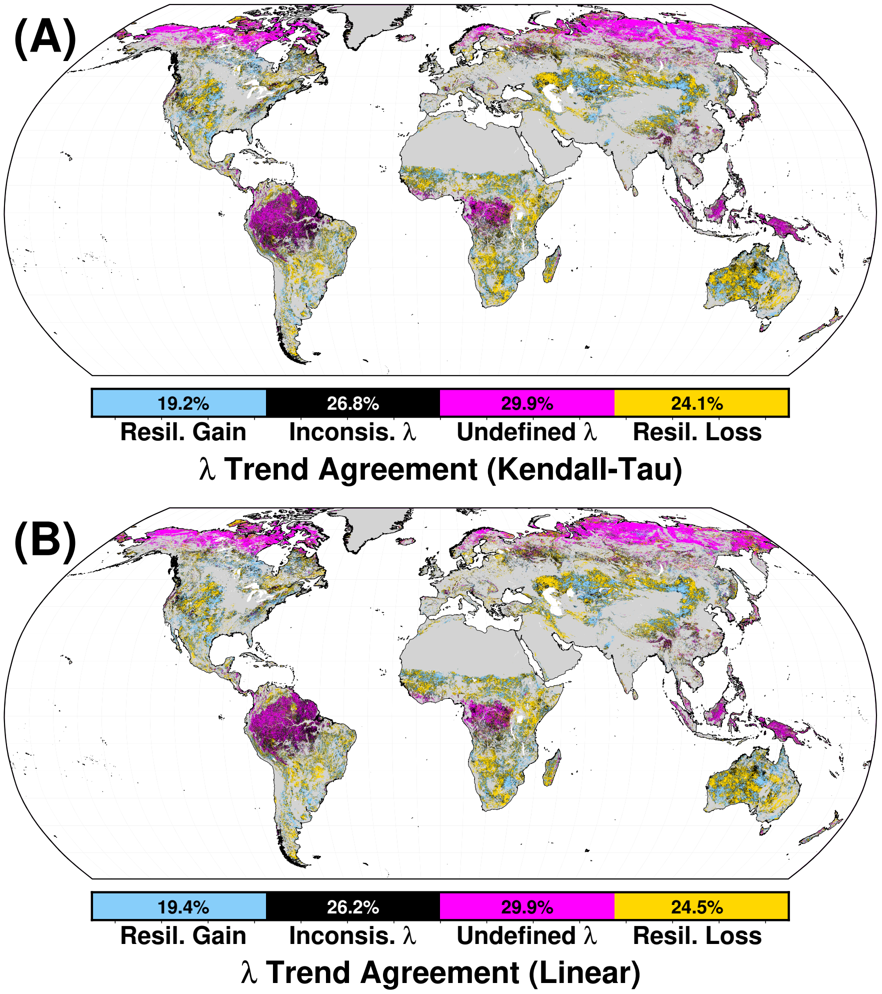

Corrected Figure ED4/ED5: Global Kendall-Tau (A) and linear (B) trends in resilience across all vegetation indices at 5 km resolution. Grey areas masked for land cover. Areas of agreement between variance- and AC1-based λ marked as resilience gain or loss, others as inconsistent (high $\lambda_{Var}/\lambda_{AC1}$ ratio, black) or undefined λ (magenta). These panels replace panel C in both Figures ED4 and ED5 of the original manuscript version.

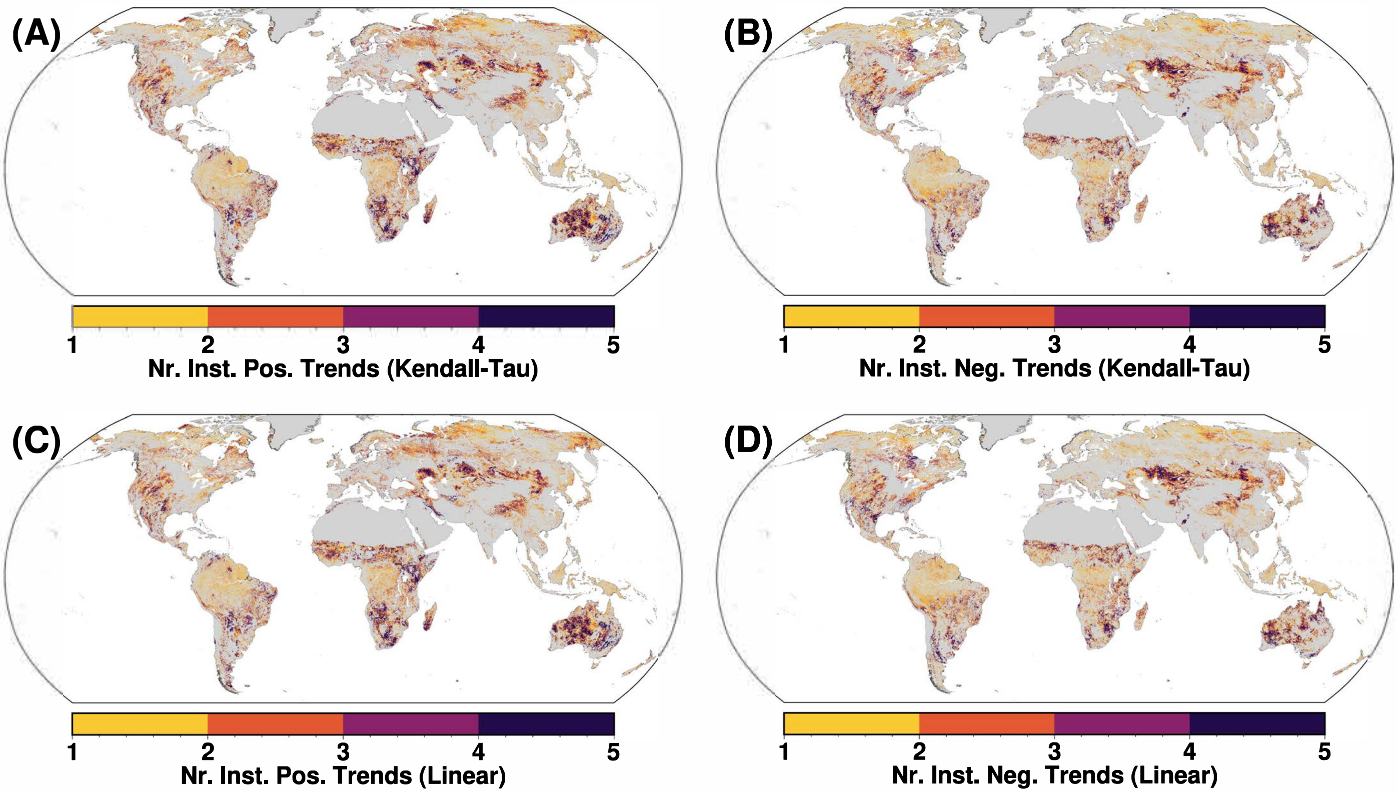

Original Extended Data Figure 6

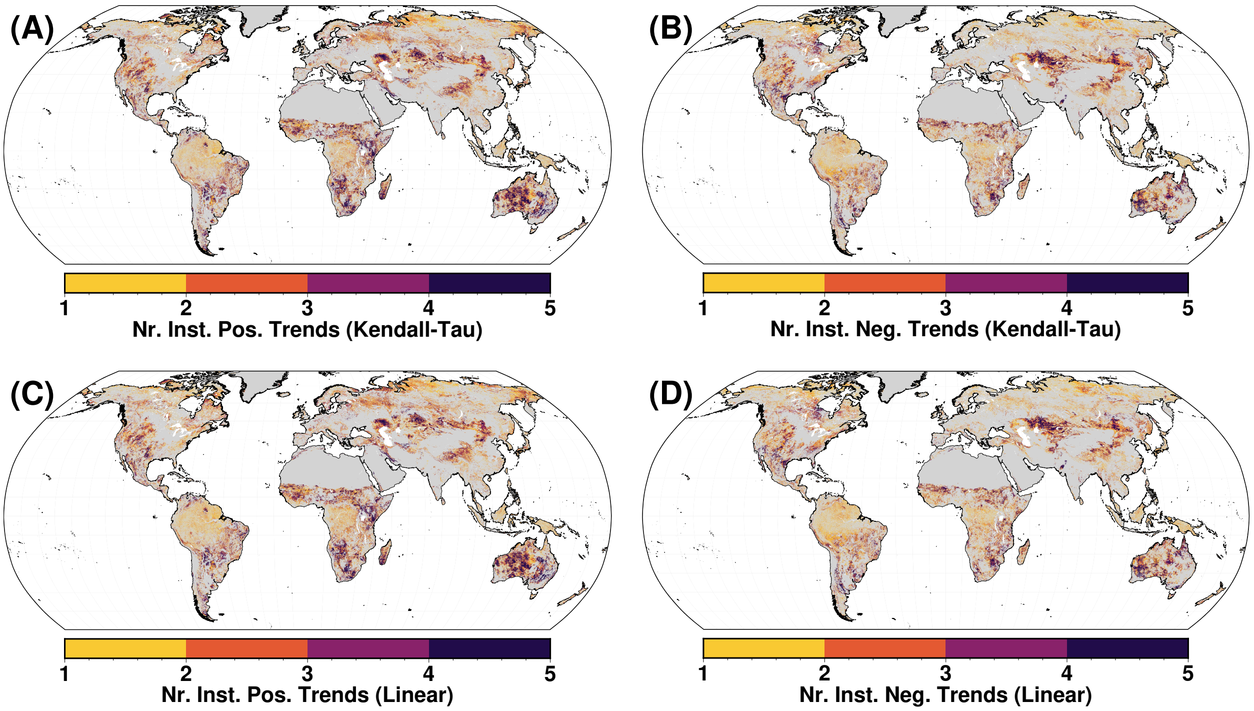

Corrected Figure ED6: Number of instruments agreeing on the direction of trend (positive/negative) for Kendall's tau statistics (A,B) and linear trends (C,D). Grey areas masked out due to anthropogenic or vegetation-free land cover. Updated from the original version (Extended Data Figure 6) to use corrected kNDVI data.

Updated Figures - Supplement

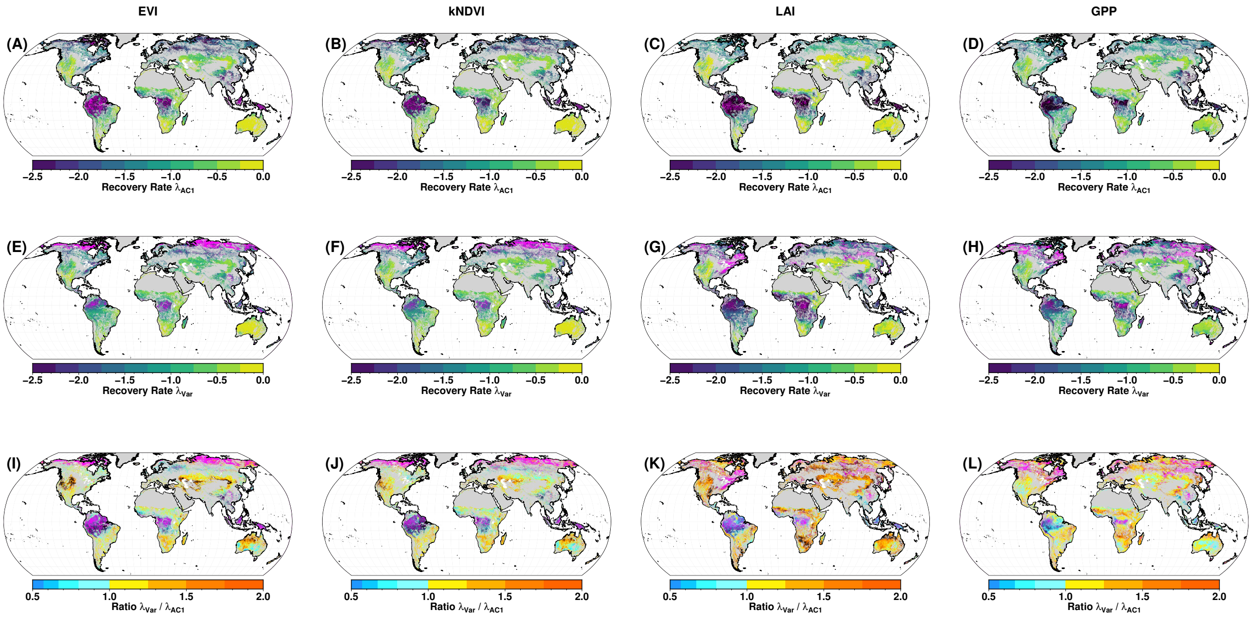

Original Supplementary Figure S7

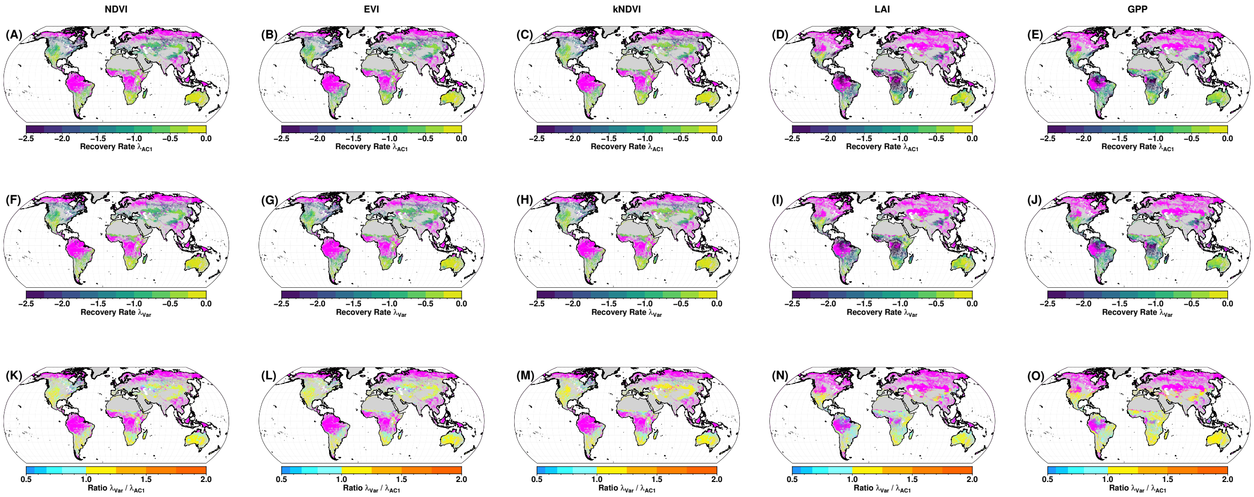

Original Supplementary Figure S8

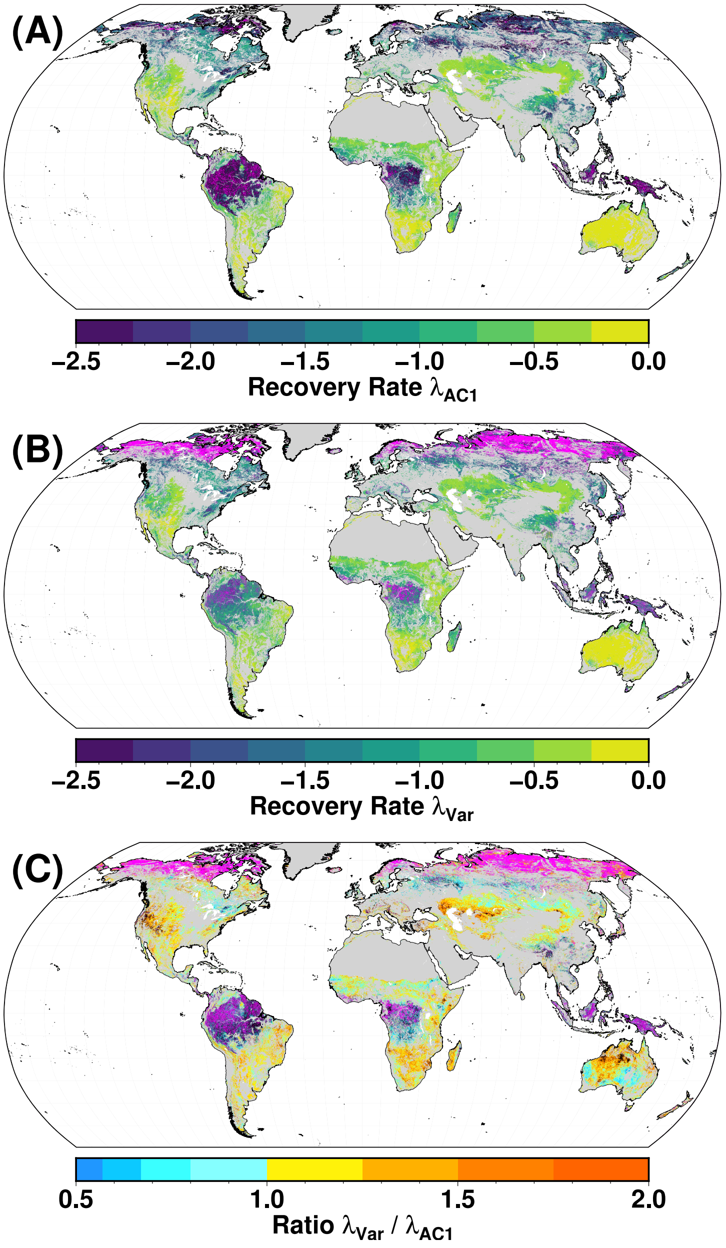

Corrected Figure S7: Comparison of global λ estimates from AC1/variance, using kNDVI. Magenta areas show unconstrained λ estimates (see Methods), black areas have a too-large $\lambda_{Var}/\lambda_{AC1}$ ratio (less than half or above a factor of two). Replaces panels B,F,J in Supplementary Figure S7 of the original supplement.

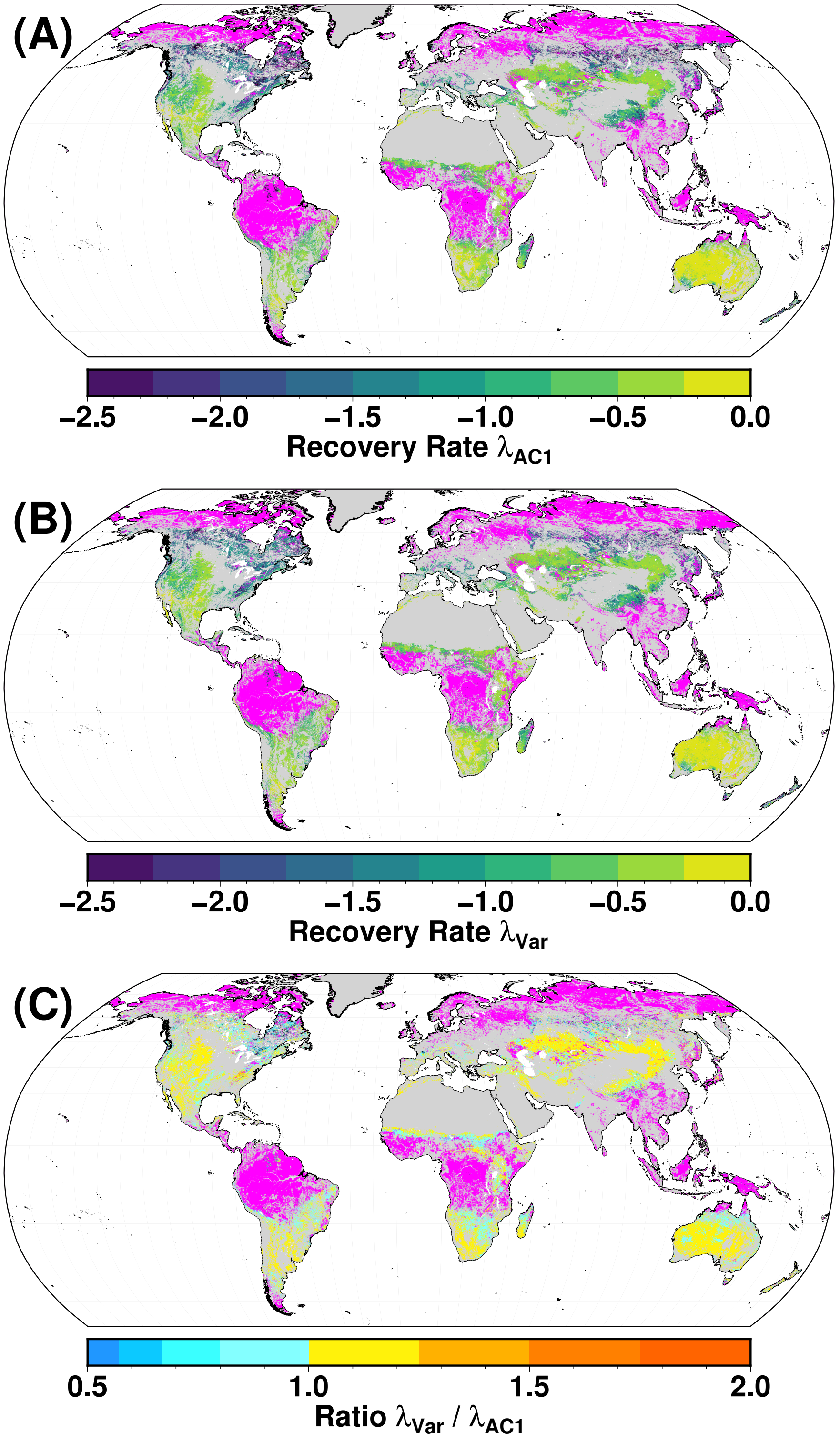

Corrected Figure S8: Comparison of global λ estimates from AC1/variance, using kNDVI; data preprocessed using STL. Magenta areas show unconstrained λ estimates, black areas have a too-large $\lambda_{Var}/\lambda_{AC1}$ ratio (less than half or above a factor of two). Replaces panels B,F,J in Supplementary Figure S8 of the original supplement.

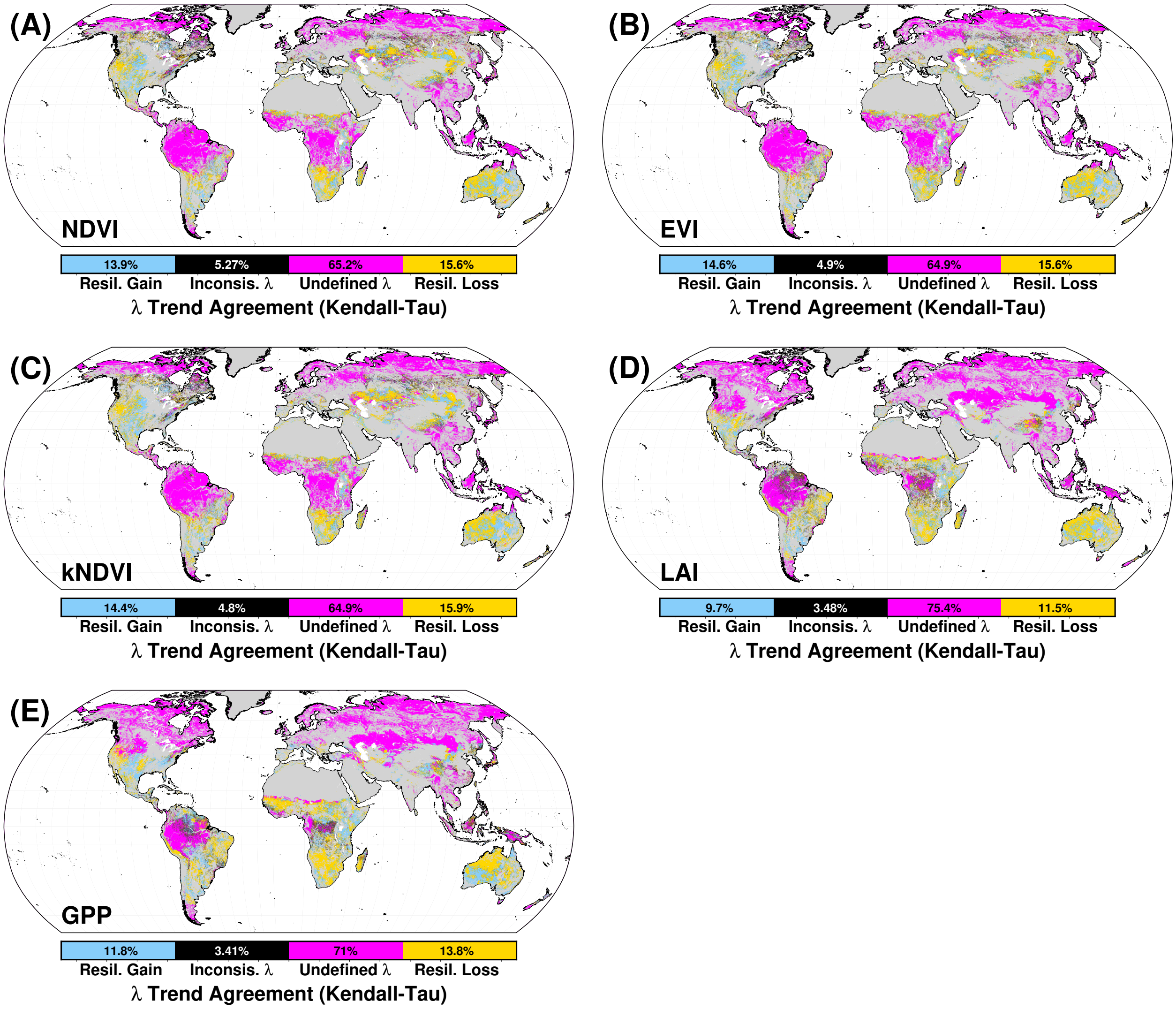

Original Supplementary Figure S10

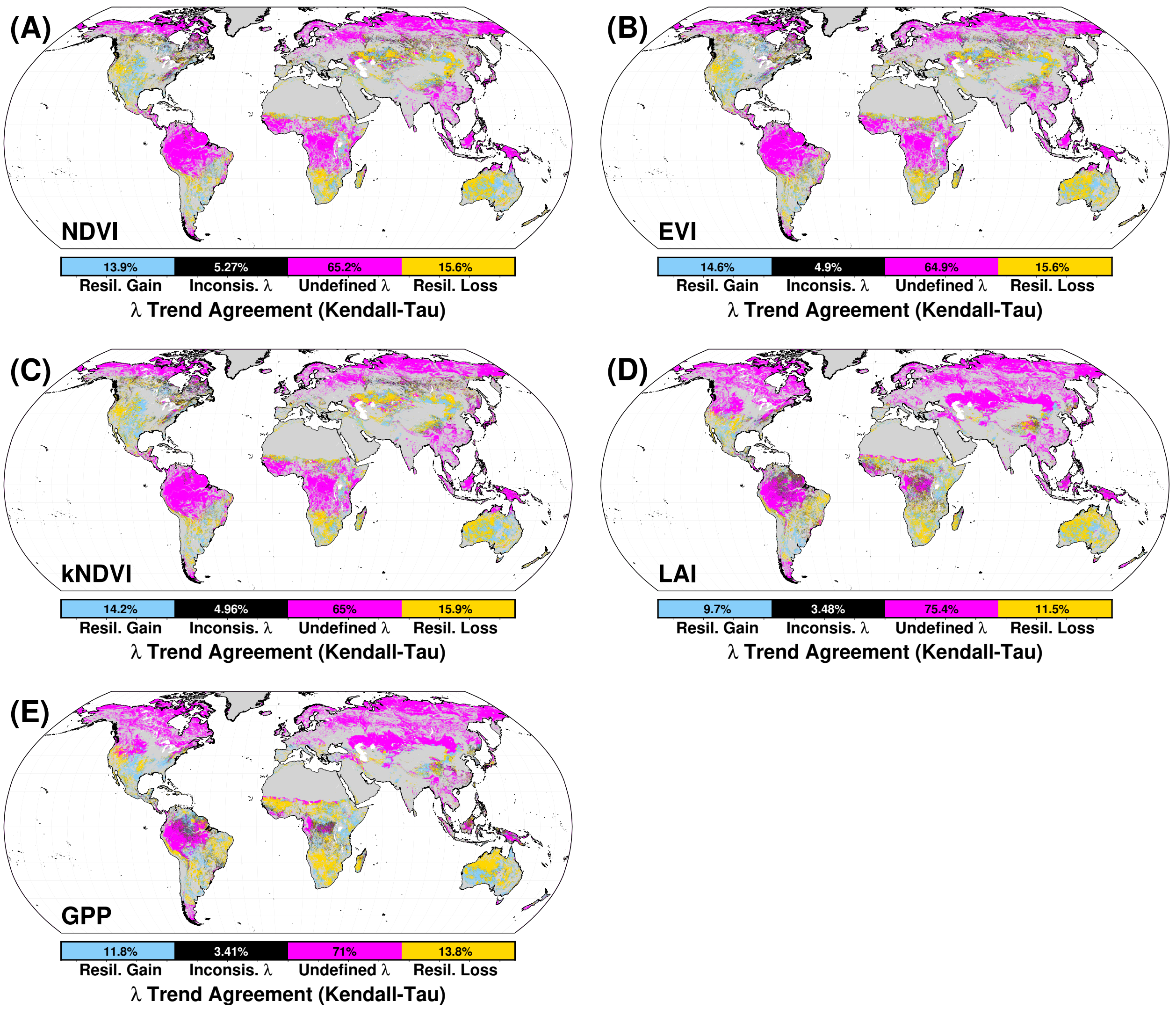

Corrected Figure S10: Global Kendall-Tau trends in resilience across all vegetation indices at 5 km resolution; data preprocessed using STL. Grey areas masked for land cover. Areas of agreement between variance- and AC1-based λ marked as resilience gain or loss, others as inconsistent (high $\lambda_{Var}/\lambda_{AC1}$ ratio, black) or undefined λ (magenta). Update to panel (C) of Supplementary Figure S10 from the original supplement.

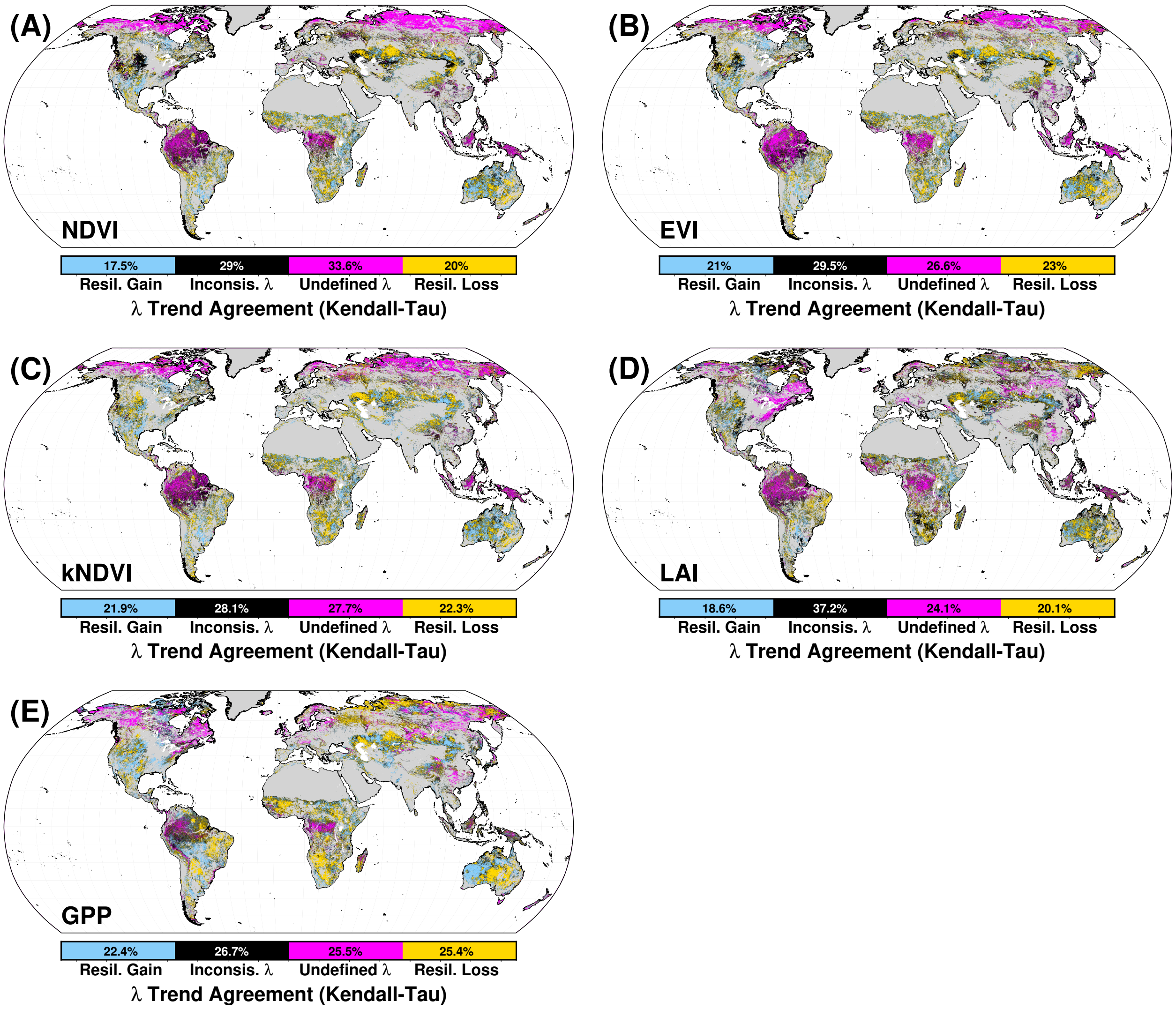

Original Supplementary Figure S13

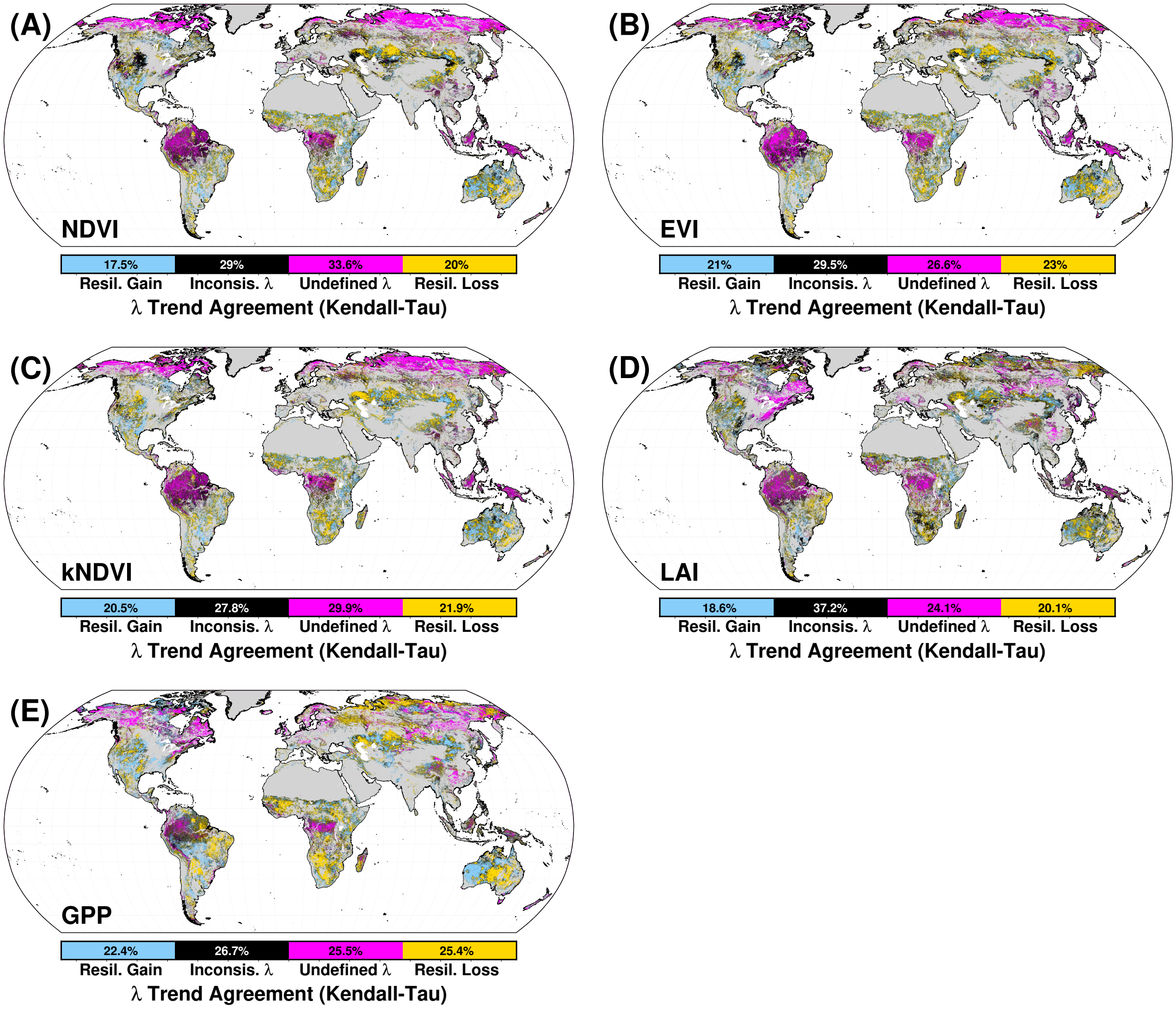

Corrected Figure S13: Global Kendall-Tau trends in resilience across all vegetation indices at 5 km resolution, over a shorter time period (2004-2017). Grey areas masked for land-cover. Areas of agreement between variance- and AC1-based λ marked as resilience gain or loss, others as inconsistent (high $\lambda_{Var}/\lambda_{AC1}$ ratio or trend disagreement, black) or undefined λ (magenta). Update to panel (C) of Supplementary Figure S13 from the original supplement.
